# Supplementary material for: From attributes to value: Neural correlates of a front-of-package label on food decision-making – An fMRI study
Source: PLoS One. 2025 Dec 5;20(12):e0336356. doi: 10.1371/journal.pone.0336356 (PMC12680182; doi:10.1371/journal.pone.0336356)
Supplement: S1 File — (DOCX) [file pone.0336356.s001.docx]

**S1 File. Preregistration and experimental design details**

**Pre-registration and minor changes**

In August 2023, the study was officially pre-registered on PsychArchives before data analysis commenced (https://doi.org/10.23668/psycharchives.13146 however, there were some deviations from the original plan. In the behavioral analysis of the main experiment, we initially planned to use Dunnett’s C test as a post-hoc test for the ANOVA. However, since Dunnett’s C test is not suitable for repeated-measures ANOVAs, we instead applied pairwise t-tests with Bonferroni correction as a more appropriate post-hoc method. For the fMRI analysis, we focused on the GLM analysis rather than the gPPI analysis. We consider the GLM analysis the first step in understanding the data and the neural mechanisms underlying the decision-making process. The gPPI analysis, along with the ROI analysis, will be the focus of the following study. Additionally, we deviated from the standardized 128 Hz high-pass filter. This decision was based on the specific nature of our experimental design, which does not follow a classical event related paradigm. We believe that a 128-second high-pass filter would not adequately account for scanner noise in our experiment due its event-related block design consisting of only two blocks, each lasting between 900 and 1100 seconds. This was an oversight in the preparation of the pre-registration and only discovered later. Therefore, we used a 2180-second high-pass filter.

**References**

Javaheri, N., Doehring, N., & Herrmann, M. (2023). Nudging Dietary Decisions with the Nutri-Score-an fMRI study.
