# Supplementary material for: From attributes to value: Neural correlates of a front-of-package label on food decision-making – An fMRI study
Source: PLoS One. 2025 Dec 5;20(12):e0336356. doi: 10.1371/journal.pone.0336356 (PMC12680182; doi:10.1371/journal.pone.0336356)
Supplement: S2 File — (DOCX) [file pone.0336356.s002.docx]

**S2 File. Stimuli**

The 63 food products were categorized into seven groups: cereals, salty snacks, sweet snacks, yogurt, nuts, canned foods, and dips. Each category contained nine products, with three assigned a green Nutri-Score, three a yellow Nutri-Score, and three a red Nutri-Score. Stimuli selection was based on familiarity and liking data from a pilot study, ensuring that only familiar and generally liked products were included. Product prices ranged from €0.55 to €3.19 (M = €2.16, SD = €0.85). When official Nutri-Scores were unavailable, they were calculated using the German government’s Nutri-Score tool (Bundesministerium für Ernährung und Landwirtschaft, April 12, 2023 under the link: <https://www.bmel.de/SharedDocs/Downloads/DE/_Ernaehrung/Lebensmittel-Kennzeichnung/nutri-score-dt-excel-berechnungstabelle.html>).

To control for whether the price of the products influenced our treatment effect, we identified all products with a real market price above €2.50, which resulted in a subset of 12 products out of the total 63. For each of these products, we calculated the average WTP across participants separately for the control and treatment conditions. The corresponding values can be found in the table and figure below. Notably, none of the products, regardless of condition, showed particularly high WTP values (see Figure 1 and Table 1 for an overview of the products).

**Figure 1**. Average WTP with SD (error bars) of all participants for products priced higher than 2.50€


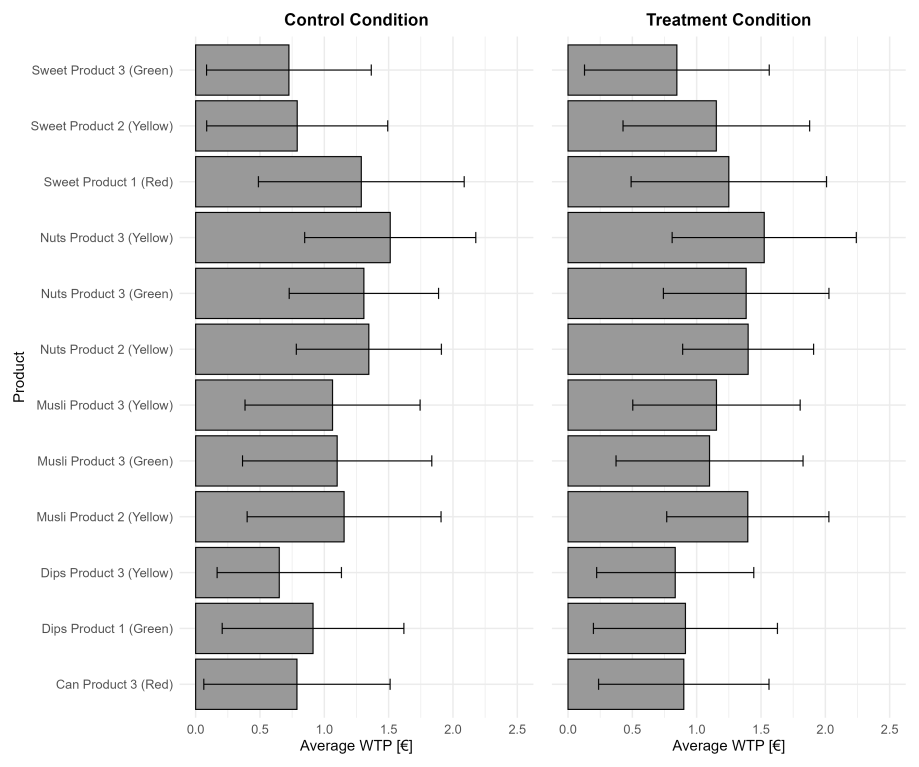


**Table 1**. Overview of products priced over 2.50€ with their price, average WTP in control and treatment conditions.

|  |  | Control | | Treatment | |
| --- | --- | --- | --- | --- | --- |
| Product | Price [€] | M | SD | M | SD |
| Can Product 3 (Red) | 2.99 | 0.79 | 0.72 | 0.9 | 0.66 |
| Dips Product 1 (Green) | 2.56 | 0.91 | 0.71 | 0.91 | 0.72 |
| Dips Product 3 (Yellow) | 3.19 | 0.65 | 0.48 | 0.83 | 0.61 |
| Cereal Product 3 (Green) | 3.79 | 1.1 | 0.74 | 1.1 | 0.73 |
| Cereal Product 2 (Yellow) | 2.69 | 1.15 | 0.75 | 1.4 | 0.63 |
| Cereal Product 3 (Yellow) | 3.79 | 1.06 | 0.68 | 1.15 | 0.65 |
| Nuts Product 3 (Green) | 2.89 | 1.31 | 0.58 | 1.38 | 0.64 |
| Nuts Product 2 (Yellow) | 3.59 | 1.35 | 0.56 | 1.4 | 0.51 |
| Nuts Product 3 (Yellow) | 3.79 | 1.51 | 0.66 | 1.52 | 0.72 |
| Sweet Product 3 (Green) | 2.65 | 0.72 | 0.64 | 0.85 | 0.72 |
| Sweet Product 1 (Red) | 2.59 | 1.29 | 0.8 | 1.25 | 0.76 |
| Sweet Product 2 (Yellow) | 2.99 | 0.79 | 0.7 | 1.15 | 0.73 |

To further examine the influence of real product prices on WTP, we conducted a mixed-effects regression analysis with participants’ WTP as the dependent variable. Product price, treatment condition, and their interaction were included as fixed effects, while participant ID and product were modeled as random effects (see Table 2). The results indicate that product price is a significant predictor of WTP. However, the interaction between price and treatment condition was not significant, suggesting that product price does not moderate the treatment effect, which is the primary focus of the study.

**Table 2.** Mixed-effects regressions predicting WTP from product price and treatment condition.

| **Predictors** | **Estimates** | **Std. Error** | ***CI*** | ***p*** |
| --- | --- | --- | --- | --- |
| Intercept | 0.75 | 0.09 | 0.57 – 0.93 | < .001 |
| Price | 0.08 | 0.04 | 0.01 – 0.15 | .032 |
| Treatment | 0.07 | 0.04 | -0.02 -0.16 | .113 |
| Price x Treatment | 0.00559 | 0.02 | -0.04 -0.05 | .794 |
| **Random effects** |  |  |  |  |
| *AIC* | 8881.893 |  |  |  |
| Conditional *R*^2^ | 0.295 |  |  |  |
| Marginal *R*^2^ | 0.012 |  |  |  |
| *ICC* | 0.286 |  |  |  |
| Observations | 4897 |  |  |  |
| *N*_Subjects_ | 40 |  |  |  |
| *N*_Products_ | 63 |  |  |  |

Product images were displayed at 30% of the screen height (324 pixels on a 1080-pixel screen) while preserving aspect ratios, except for one wider product, which was displayed at 25% height. In the treatment condition, each image was enclosed by a 5 pixel thick colored frame (green, yellow, or red), representing the Nutri-Score category.

**References**

Bundesministerium für Ernährung und Landwirtschaft. (2023, April 12). Nutri-Score: Berechnungstabelle (Excel). https://www.bmel.de/SharedDocs/Downloads/DE/_Ernaehrung/Lebensmittel-Kennzeichnung/nutri-score-dt-excel-berechnungstabelle.html
