# Supplementary material for: From attributes to value: Neural correlates of a front-of-package label on food decision-making – An fMRI study
Source: PLoS One. 2025 Dec 5;20(12):e0336356. doi: 10.1371/journal.pone.0336356 (PMC12680182; doi:10.1371/journal.pone.0336356)

**S3 File Response time analysis**

**Analysis of reaction time**

To analyze the effects of frame condition (control vs. treatment) and Nutri-Level (green, yellow, red) on reaction times (see Fig 1), we conducted a repeated-measures analysis of variance (ANOVA), treating participant ID as a random factor. A significant effect of treatment condition was observed (F(1,39) = 54.11, p < .001) indicating that reaction times were significantly shorter in the treatment condition (M = 2.25 s) compared to the control condition (M = 2.47 s). This decrease in reaction time in the treatment condition compared to the control conditions can have two different reasons. One, it might be because of a learning effect, as the control trials were always presented first and followed by the treatment trials. Second, it might be that the color-coded frame is seen as a heuristic, so that participants can evaluate the products faster by considering the frame. The main effect of frame color was significant (F(2,78) = 4.40, p = .016), suggesting that mean reaction times varied across Nutri-Level categories (Green: M= 2.38s; Yellow: M = 2.37s; Red: M = 2.33s ). The interaction between treatment condition and frame color was not significant (F(2,78) = 2.59, p = .081), suggesting that the effect of treatment condition on reaction times was independent of frame color.

**Fig 1**

Average reaction time over all trials
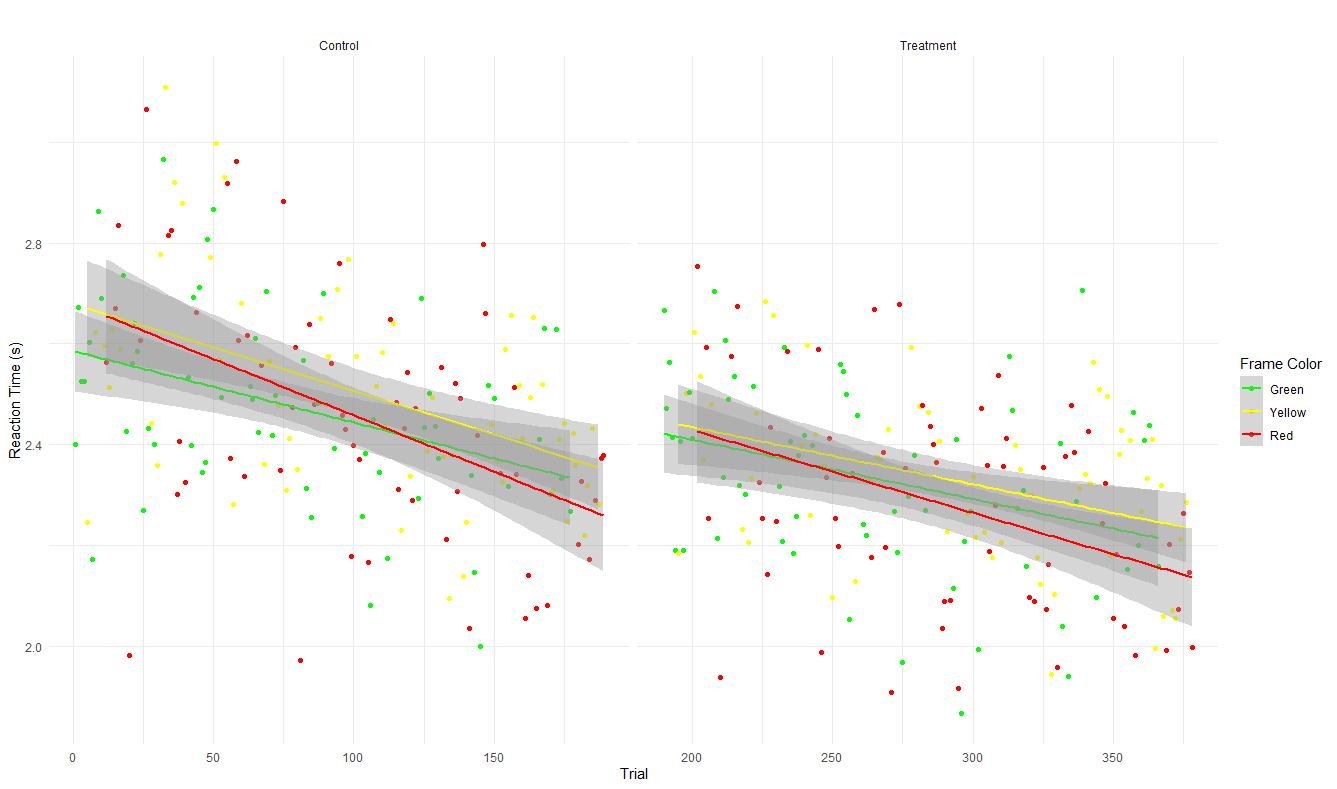

Supplement: S3 File — (DOCX) [file pone.0336356.s003.docx]
