# Supplementary material for: From attributes to value: Neural correlates of a front-of-package label on food decision-making – An fMRI study
Source: PLoS One. 2025 Dec 5;20(12):e0336356. doi: 10.1371/journal.pone.0336356 (PMC12680182; doi:10.1371/journal.pone.0336356)
Supplement: S4 File — (DOCX) [file pone.0336356.s004.docx]

**S4 File Post-hoc questionnaire analysis**

In the post-hoc questionnaire participants were asked to re-rate the products, answering the question how much they are willing to pay for each product, and how much they are willing to pay for the product with the information of its real Nutri-Score on a continuous scale. Average WTP of participants in the fMRI and in the post-hoc questionnaire were compared to investigate whether average responses differed between the MRI setting and in the post-hoc questionnaire. A repeated-measure ANOVA was conducted using average responses of participants per setting, treatment condition, and color-coded frame. The Shapiro–Wilk test indicated that all variables were normally distributed (all p-values > 0.1), and no outliers were identified. The results of the ANOVA indicated a significant main effect of setting (*F* (1, 39) = 8.84, *p* = .005, *η²* = .040), main effect of treatment (*F* (1, 39) = 28.57, *p* < .001, *η²* = .008), and main effect in the color-code (*F* (2, 78) = 56.84, *p* < .001, *η²* = .094). Thus, average WTP differed between settings, between treatment, and color-codes, with a significant interaction effect between setting and treatment (*F* (1, 39) = 6.63, *p* = .014, *η²* = .002), a significant interaction effect between treatment and color-coded frame (*F* (2, 78) = 52.13, *p* < .001, *η²* = .019), as well as a significant three-way interaction between setting, treatment condition, and color-coded frame (*F* (2, 78) = 7.75, *p* = .001, *η²* = .002). The results suggest that there is a variation between how setting and treatment condition influences across color-coded frames. Furthermore, post-hoc paired t-tests were conducted within each color-coded frame and treatment condition to compare settings, with Bonferroni correction applied. For average WTP across settings, treatment conditions, and color-coded frames, see Fig 1.

**Fig 1**

Average WTP per Setting, Treatment and Color-Coded Frame Condition


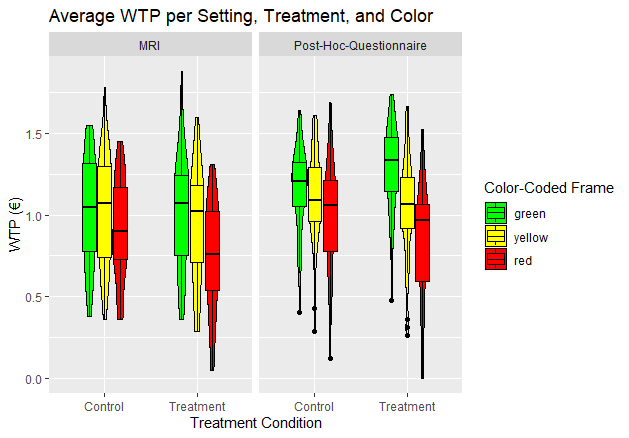


In both the control condition and treatment conditions, average WTP was significantly higher in the post-hoc-questionnaire setting (*M_post-hoc-control_* = 1.09, *SD_post-hoc-control_* = 0.31; *M_post-hoc-treatment_* = 1.06, *SD_post-hoc-treatment_* = 0.37) compared to the MRI setting (*M_MRI-control_* = 0.99, *SD_MRI-control_* = 0.32; *p_Bonf_* < .05; *M_MRI-treatment_* = 0.90, *SD_MRI-treatment_* = 0.36; *p_Bonf._* < .001). A significant difference in WTP between settings was observed for the green color-coded frame in both the control (*p_Bonf.-_* < .05) and treatment conditions (*p_Bonf._* < .001). Participants in the post-hoc-questionnaire condition reported higher WTP (*M_post-hoc-treatment-green_* = 1.28, *SD_post-hoc-treatment-green_* = 0.28; *M_post-hoc-control-green_* = 1.16, *SD_post-hoc-control-green_* = 0.26) than in the MRI condition (*M_MRI-treatment -green_*= 1.02, *SD_MRI-treatment-green_* = 0.35; *M_MRI-control-green_* = 1.03, *SD_MRI-control-green_* = 0.33). In contrasts, no significant differences between settings were found for the yellow color-coded frame (*p_Bonf._*= .29 for control condition; *p_Bonf._*= .21 for treatment condition). Although WTP was higher in the post-hoc-questionnaire setting (*M_post-hoc-treatment-yellow_* = 1.04, *SD_post-hoc-treatment-yellow_* = 0.32, *M_post-hoc-control-yellow_* = 1.10, *SD_post-hoc-control-yellow_* = 0.30) relative to the MRI setting (*M_MRI-treatment -yellow_* = 0.95, *SD_MRI-treatment-yellow_* = 0.34; *M_MRI-control-yellow_* = 1.03*, SD_MRI-control-yellow_* = 0.33), the differences were not statistically significant. Similarly, for the red color-coded frame, no significant setting-related difference was found (*p_Bonf._* = .18 for treatment condition; *p_Bonf._* = .17 for control condition). As with the yellow condition, average WTP was higher in the post-hoc questionnaire setting (*M_post-hoc-treatment-red_* = 0.85, *SD_post-hoc-treatment-red_* = 0.36; *M_post-hoc-control-red_* = 1.00, *SD_post-hoc-control-red_* = 0.35) compared to the MRI condition (*M_MRI-treatment -red_* = 0.74, *SD_MRI-treatment-red_* = 0.33*; M_MRI-control-red_* = 0.90, *SD_MRI-control-red_* = 0.30).

Taken together, these results indicate that while no significant treatment effects were observed for the yellow and red color-coded conditions, a significant difference emerged for the green condition between the settings.
