# Supplementary material for: From attributes to value: Neural correlates of a front-of-package label on food decision-making – An fMRI study
Source: PLoS One. 2025 Dec 5;20(12):e0336356. doi: 10.1371/journal.pone.0336356 (PMC12680182; doi:10.1371/journal.pone.0336356)
Supplement: S5 File — (DOCX) [file pone.0336356.s005.docx]

**S5 File Motion detection estimation**

**Additional Motion Detection**

Net movement between consecutive volumes was approximated using the formula:

$$\sqrt{\left( \Delta_{x} \right)^{2}+{\left( \Delta_{y} \right)^{2}+\left( \Delta_{z} \right)}^{2}+ 58*\left( \left( r_{x} \right)^{2}+\left( r_{y} \right)^{2}+\left( r_{z} \right)^{2} \right)}$$

, where$\Delta_{x}$,$\Delta_{y}$, and $\Delta_{z}$ represent translational motion parameters, and $r_{x}$, $r_{y}$ , and $r_{z}$ represent rotational motion parameters along the x, y, and z axes. Volumes with a net motion exceeding 0.3 mm were flagged, and the total number of flagged volumes was calculated for each participant. Participants with flagged volumes exceeding 5% of the total were excluded from the analysis. No participant exceeded this criterion.
