# Supplementary material for: From attributes to value: Neural correlates of a front-of-package label on food decision-making – An fMRI study
Source: PLoS One. 2025 Dec 5;20(12):e0336356. doi: 10.1371/journal.pone.0336356 (PMC12680182; doi:10.1371/journal.pone.0336356)
Supplement: S6 File — (DOCX) [file pone.0336356.s006.docx]

**S6 File Multilevel analysis of behavioral data**

Linear mixed-effects models for all key dependent variables (WTP, perceived healthiness, and perceived tastiness) were conducted. These models account for trial-level variability by including random intercepts for both participants (id) and stimuli (product).Thus, the models control for between-subject and between-stimulus differences addressing the hierarchical nature of the data and the potential presence of missing responses. Overall, the results are consistent with the behavioral findings reported in the paper using the preregistered ANOVA, indicating that including products as a random effect did not substantially alter the pattern of results. In the following, the results of the analysis can be found.

- WTP

The mixed model revealed significant main effects of color-coded frame (*F* (2, 60.1) = 5.74, *p* = .005) and Treatment (*F* (1, 4854.6) = 16.27, *p* < .001), as well as a significant interaction (*F* (2, 4854.6) = 4.75, *p* = .0087). Planned contrasts comparing Control vs. Treatment within each frame color yielded can be found under Table 1.

**Table 1.**

Contrasts of WTP Between Treatment and Control Conditions Within Each Frame Color.

| Contrast | Estimate | SE | z | p |
| --- | --- | --- | --- | --- |
| Green - Control vs Treatment | 0.009 | 0.030 | 0.292 | .770 |
| Yellow - Control vs Treatment | 0.062 | 0.030 | 2.07 | .039 |
| Red - Control vs Treatment | 0.139 | 0.030 | 4.63 | < .001 |

These results indicate that Treatment significantly reduced WTP for red- and yellow-framed products, but not for green-framed products.

- Healthiness Perception

The model showed a significant main effect of color-coded frame (*F* (2, 60.0) = 12.92, *p* < .001) and a strong interaction with Treatment (*F* (2, 4840.2) = 113.39, *p* < .001). The main effect of Treatment was not significant (*F* (1, 4840.2) = 3.43, *p* = .064). The planned contrasts of healthiness perception can be seen in Table 2.

**Table 2.**

Contrasts of Healthiness Perception Between Treatment and Control Conditions Within Each Frame Color.

| Contrast | Estimate | SE | z | p |
| --- | --- | --- | --- | --- |
| Green - Control vs Treatment | –0.436 | 0.048 | –9.03 | < .001 |
| Yellow - Control vs Treatment | 0.004 | 0.048 | 0.090 | .928 |
| Red - Control vs Treatment | 0.586 | 0.048 | 12.20 | < .001 |

These results show that the treatment effect strongly depended on frame color: participants rated red-framed products as significantly less healthy in the treatment condition, while the green frame led to higher perceived healthiness under treatment. No significant difference was found in the yellow condition.

- Tastiness Perception

The model revealed significant effects of color-coded frame (*F* (2, 60.1) = 3.83, *p* = .027), Treatment (*F* (1, 4848.4) = 12.07, *p* < .001), and a significant interaction (*F* (2, 4848.4) = 3.29, *p* = .037). The planned contrasts for the decision domain tastiness perception can be found under Table 3.

**Table 3.**

Contrasts of Tastiness Perception Between Treatment and Control Conditions Within Each Frame Color.

| Contrast | Estimate | SE | z | p |
| --- | --- | --- | --- | --- |
| Green - Control vs Treatment | 0.001 | 0.065 | 0.021 | .983 |
| Yellow - Control vs Treatment | 0.158 | 0.065 | 2.41 | .016 |
| Red - Control vs Treatment | 0.234 | 0.065 | 3.58 | < .001 |

These findings suggest that tastiness ratings showed a significant decrease in the treatment condition for red and yellow-framed products, while no change was observed in the green condition.
