# Supplementary material for: From attributes to value: Neural correlates of a front-of-package label on food decision-making – An fMRI study
Source: PLoS One. 2025 Dec 5;20(12):e0336356. doi: 10.1371/journal.pone.0336356 (PMC12680182; doi:10.1371/journal.pone.0336356)
Supplement: S7 File — (DOCX) [file pone.0336356.s007.docx]

**S7 File Parametric modulation analysis with WTP**

Previous studies have suggested a relationship between WTP and vmPFC (Plassmann et al., 2007; Newton-Fenner et al., 2023). To replicate this finding, we conducted a parametric modulation analysis within the original general linear model, which included regressors for each condition, as well as parametric modulators for WTP, healthiness, and tastiness ratings. All parametric modulators were demeaned to account for interindividual differences. To specifically assess neural sensitivity to WTP, we specified a contrast averaging the six WTP parametric modulators (each weighted 1/6) against a baseline of zero. At the second level, we applied a cluster-level family-wise error (FWE) correction at p < 0.05, using an uncorrected voxel-level threshold of p < 0.001 (two-sided).

Our data (Figure 2, Table 2) revealed significant correlation between WTP and dorsal anterior cingulate cortex (dACC; BA32) and ventromedial prefrontal cortex (vmPFC; BA11). These findings are in line with a recent meta-analysis by Newton-Fenner et al. (2023), who identified consistent WTP-related activation in the left orbitofrontal cortex (OFC; BA11) and the left vmPFC (BA10/BA32) using the Becker–DeGroot–Marschak (BDM) auction method

Figure 1. Neural correlates of WTP


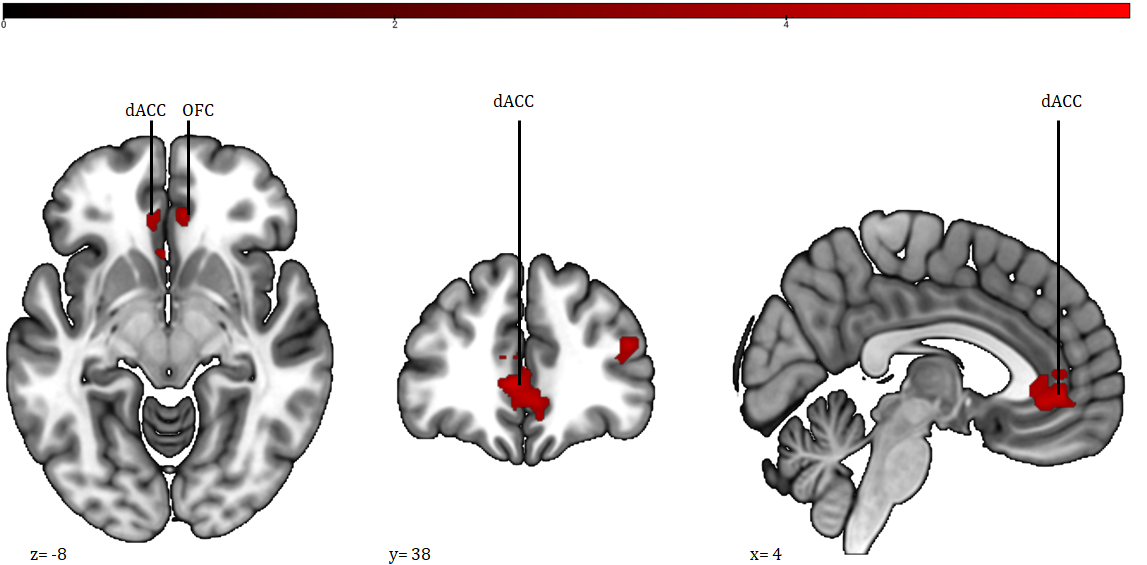


The t-map is projected onto an MNI_152_2009 template. A p -threshold of < .001 (two-sided) and a FWE-cluster threshold of p<.05 (df = [1, 39]) was used. Abbreviations in the figure: dACC = dorsal anterior cingulate cortex (BA32); OFC =orbital frontal cortex (BA11). Color bar indicates t-values.

Table 1. Neural correlates of WTP

| Cluster Nr. | Hemisphere | Brodmann  Area | Peak | x | y | z | Peak t Score | Cluster Size (k) |
| --- | --- | --- | --- | --- | --- | --- | --- | --- |
| 1 | L | 24 | Ventral Anterior Cingulate Cortex | -4 | 30 | 2 | 5.86 | 645 |
|  | L | 11 | Orbitofrontal Cortex | -6 | 26 | -14 | .5.67 |  |
|  | R | 32 | Dorsal Anterior Cingulate Cortex | 10 | 32 | 4 | 5.36 |  |
|  | L | 32 | Dorsal Anterior Cingulate Cortex | -2 | 42 | 2 | 4.75 |  |
| 2 | R | / | Putamen | 30 | 12 | 2 | 5.26 | 253 |
|  | R | 13 | Insula | 38 | 16 | 2 | 4.15 |  |
| 3 | L | 40 | Supramarginal Gyrus | -52 | -44 | 54 | 5.19 | 105 |
|  | L | 7 | Visual Motor Area | -40 | -44 | 48 | 4.72 |  |
|  | L | 39 | Angular Gyrus | -42 | -46 | 40 | 4.68 |  |
| 4 | R | 46 | Dorsolateral Prefrontal Cortex (lateral part) | 42 | 44 | 8 | 5.05 | 100 |
|  | R | 9 | Dorsolateral Prefrontal Cortex (dorsal part) | 44 | 38 | 20 | 4.50 |  |

Threshold T = 3.56, p uncorrected (two-sided, voxel/peak level) < .001, cluster defining threshold (cluster size, in voxels) => 100 voxels, p FWE corrected (cluster level) < .05, df = [1,39]. Only unidirectional effects were found. Cluster size is displayed in number of voxels. The table shows additional local maxima more than 4.0 mm apart. Clusters with multiple peaks in the same brain region are only reported once. L = Left; R = Right.

**References**

Newton-Fenner, A., Hewitt, D., Henderson, J., Roberts, H., Mari, T., Gu, Y., ... & Stancak, A. (2023). Economic value in the Brain: A meta-analysis of willingness-to-pay using the Becker-DeGroot-Marschak auction. Plos one, 18(7), e0286969.

Plassmann, H., O'doherty, J., & Rangel, A. (2007). Orbitofrontal cortex encodes willingness to pay in everyday economic transactions. Journal of neuroscience, 27(37), 9984-9988.
