# Supplementary material for: From attributes to value: Neural correlates of a front-of-package label on food decision-making – An fMRI study
Source: PLoS One. 2025 Dec 5;20(12):e0336356. doi: 10.1371/journal.pone.0336356 (PMC12680182; doi:10.1371/journal.pone.0336356)
Supplement: S1 Table — (DOCX) [file pone.0336356.s008.docx]

**S1 Table. Conjunction of the contrasts according to a logical “and” calculation: WTP (treatment > control), healthiness (treatment > control), tastiness (treatment > control)**

| **Cluster Nr.** | **Hemisphere** | **Brodmann Area** | **Peak** | **x** | **y** | **z** | **Peak *t* Score** |
| --- | --- | --- | --- | --- | --- | --- | --- |
| 1 | R | BA46 | Lateral Dorsolateral Prefrontal Cortex | 42 | 38 | 0 | 2584 |
| 2 | L | BA11 | Orbital Frontal Cortex | -12 | 30 | -8 | 1290 |
| 3 | R | BA20 | Inferior Temporal Gyrus | 50 | -34 | -16 | 972 |
| 4 | R | BA19 | Visual Association Cortex | 40 | -82 | 16 | 624 |
| 5 | R | BA37 | Fusiform | 34 | -50 | -20 | 529 |
| 6 | L | BA21 | Medial Temporal Gyrus | -64 | -22 | -16 | 474 |
| 7 | R | BA38 | Temporal Pole | 54 | 8 | -34 | 200 |
| 8 | L | BA7 | Visual Motor | -26 | -54 | 40 | 186 |
| 9 | R | BA31 | Dorsal Posterior Cingulate Cortex | 6 | -48 | 42 | 158 |
| 10 | R | BA37 | Fusiform | 48 | -54 | -26 | 148 |
| 11 | R | BA7 | Visual Motor | 2 | -74 | 38 | 102 |
| 12 | R | BA9 | Dorsal Dorsolateral Prefrontal Cortex | 4 | 46 | 36 | 100 |
| 13 | L | BA23 | Ventral Posterior Cingulate Cortex | -2 | -44 | 22 | 93 |
| 14 | R | - | Putamen | 24 | 22 | -2 | 30 |
| 15 | L | BA20 | Inferior Temporal Gyrus | -52 | -26 | -22 | 24 |
| 16 | R | BA11 | Orbital Frontal Cortex | 14 | 32 | -8 | 21 |

*Note.* Threshold *T* = 3.56, *p* _uncorrected_ (two-sided, voxel/peak level) < .001, *p _FWE_* _corrected_ (cluster level) < .05, df = [1,39], for all three contrasts. No regions showed higher activation in control than treatment and only unidirectional effects were found. As the results show a conjunction analysis, no t-score are reported. All clusters are k > 20
