# Supplementary material for: From attributes to value: Neural correlates of a front-of-package label on food decision-making – An fMRI study
Source: PLoS One. 2025 Dec 5;20(12):e0336356. doi: 10.1371/journal.pone.0336356 (PMC12680182; doi:10.1371/journal.pone.0336356)
Supplement: S2 Table — (DOCX) [file pone.0336356.s009.docx]

**S2 Table. Brain regions showing significant activation for the contrast treatment > control during WTP ratings.**

| **Cluster Nr.** | **Hemisphere** | **Brodmann Area**  **Area** | **Peak** | **x** | **y** | **z** | **Peak *t* score** | **Cluster size (k)** |
| --- | --- | --- | --- | --- | --- | --- | --- | --- |
| 1 | R | BA10 | Anterior prefrontal | 20 | 62 | 16 | 6.56 | 2919 |
|  | R | BA10 | Anterior prefrontal cortex | 30 | 60 | 16 | 5.86 |  |
|  | R | BA44 | Inferior Frontal Gyrus | 42 | 10 | 30 | 5.74 |  |
| 2 | R | BA37 | Fusiform | 32 | -42 | -14 | 6.93 | 2337 |
|  | R | BA21 | Medial Temporal Gyrus | 46 | -36 | 2 | 6.63 |  |
|  | R | BA21 | Medial Temporal Gyrus | 60 | -44 | 2 | 6.18 |  |
| 3 | L | BA46 | Lateral Dorsolateral prefrontal cortex | -28 | 40 | 6 | 5.71 | 1563 |
|  | L | BA9 | Dorsal Dorsolateral prefrontal cortex | -38 | 26 | 22 | 5.63 |  |
|  | L | BA45 | Inferior Frontalal Gyrus | -28 | 30 | 12 | 5.53 |  |
| 4 | R | BA39 | Angular Gyrus | 30 | -66 | 42 | 5.62 | 710 |
|  | R | BA39 | Angular Gyrus | 32 | -68 | 32 | 4.99 |  |
|  | R | BA7 | Visual-Motor | 32 | -50 | 46 | 4.67 |  |
| 5 | L | BA37 | Fusiform | -36 | -44 | -14 | 5.47 | 525 |
|  | L | BA37 | Fusiform | -28 | -56 | -10 | 5.27 |  |
|  | L | BA37 | Fusiform | -36 | -52 | -10 | 4.79 |  |
| 6 | L | BA21 | Medial Temporal Gyrus | -56 | -42 | -2 | 5.80 | 519 |
|  | L | BA21 | Medial Temporal Gyrus | -50 | -30 | -8 | 5.70 |  |
|  | L | BA21 | Medial Temporal Gyrus | -66 | -32 | -8 | 5.42 |  |

*Note.* (*T* = 3.56, *p_uncorrected_* < .001, two-sided, voxel/peak level. The cluster-defining threshold was set at *k* ≥ 96 voxels, with an *FWE*-corrected significance level of *p* < .001 (cluster level), *df* = [1,39]. No regions exhibited greater activation in the control > treatment contrast. All coordinates are reported in MNI space, and cluster size is given in voxel count.
