# Supplementary material for: From attributes to value: Neural correlates of a front-of-package label on food decision-making – An fMRI study
Source: PLoS One. 2025 Dec 5;20(12):e0336356. doi: 10.1371/journal.pone.0336356 (PMC12680182; doi:10.1371/journal.pone.0336356)
Supplement: S3 Table — (DOCX) [file pone.0336356.s010.docx]

**S3 Table. Brain regions showing significant activation in the treatment > control contrast during healthiness ratings.**

| **Cluster Nr.** | **Hemisphere** | **Brodmann Area**  **Area** | **Peak** | **x** | **y** | **z** | **Peak *t* Score** | **Cluster Size (*k*)** |
| --- | --- | --- | --- | --- | --- | --- | --- | --- |
| 1 | L | BA21 | Medial Temporal Gyrus | -58 | -42 | -2 | 11.30 | 43389 |
|  | R | BA21 | Medial Temporal Gyrus | 64 | -40 | -2 | 11.08 |  |
|  | L | BA39 | Angular Gyrus | -42 | -56 | 42 | 10.63 |  |
| 2 | R | BA30 | Agranular Retrolimb | 4 | -46 | 8 | 5.34 | 345 |
|  | R | BA36 | Parahippocampus | 8 | -42 | 2 | 4.94 |  |
|  | L | BA19 | Visual Association Cortex | -8 | -46 | -6 | 4.67 |  |
| 3 | R | - | Thalamus | 12 | -4 | 4 | 5.72 | 94 |
|  | R | - | Thalamus | 18 | -8 | 8 | 4.21 |  |

*Note.* (*T* = 3.56, *p_uncorrected_* < .001, two-sided, voxel/peak level). The cluster-defining threshold was set at *k* ≥ 94 voxels, with a family-wise error (*FWE*) cluster-level correction (*p* < .05), df = [1, 39]. No regions exhibited greater activation in the control > treatment contrast. All coordinates are reported in MNI space, and cluster size is given in voxel count. The lower section of the table presents activation clusters surviving an FWE voxel-level correction (>.05), with an extent threshold of *k* ≥ 20 voxels.
