# Supplementary material for: From attributes to value: Neural correlates of a front-of-package label on food decision-making – An fMRI study
Source: PLoS One. 2025 Dec 5;20(12):e0336356. doi: 10.1371/journal.pone.0336356 (PMC12680182; doi:10.1371/journal.pone.0336356)
Supplement: S4 Table — (DOCX) [file pone.0336356.s011.docx]

**S4 Table. Brain regions showing significant activation in the treatment > control contrast during tastiness ratings.**

| **Cluster Nr.** | **Hemisphere** | **Brodmann Area**  **Area** | **Peak** | **x** | **y** | **z** | **Peak *t* Score** | **Cluster Size (*k*)** |
| --- | --- | --- | --- | --- | --- | --- | --- | --- |
| 1 | R | BA21 | Medial Temporal Gyrus | 62 | -26 | -8 | 9.47 | 32699 |
|  | R | BA22 | Superior Temporal Gyrus | 64 | -38 | 0 | 8.48 |  |
|  | R | BA10 | Anterior Prefrontal Cortex | 40 | 50 | 22 | 7.91 |  |
| 2 | L | BA21 | Medial Temporal Gyrus | -60 | -44 | -2 | 7.11 | 1259 |
|  | L | BA37 | Fusiform | -58 | -52 | -2 | 6.83 |  |
|  | L | BA21 | Medial Temporal Gyrus | -66 | -32 | -8 | 6.77 |  |
| 3 | L | - | Cerebellum | -12 | -84 | -28 | 7.85 | 1487 |
|  | - | BA18 | Secondary Visual Cortex | 0 | -82 | -4 | 6.48 |  |
|  | R | - | Cerebellum | 4 | -92 | -10 | 5.82 |  |
| 4 | L | BA19 | Visual Association Cortex | -28 | -50 | -6 | 4.43 | 91 |
|  | L | BA37 | Fusiform | -26 | -58 | -10 | 4.20 |  |
|  | L | BA19 | Visual Association Cortex | -28 | -56 | 0 | 3.88 |  |

*Note.* (*T* = 3.56, *p_uncorrected_* < 0.001, two-sided, voxel/peak level). The cluster-defining threshold was set at *k* ≥ 91 voxels, with a family-wise error (*FWE*) cluster-level correction (*p* < .001), df = [1,39]. No regions exhibited greater activation in the control > treatment contrast. All coordinates are reported in MNI space, and cluster size is given in voxel count. The lower section of the table presents activation clusters surviving an FWE voxel-level correction, with an extent threshold of *k* ≥ 20 voxels.
