# Supplementary material for: From attributes to value: Neural correlates of a front-of-package label on food decision-making – An fMRI study
Source: PLoS One. 2025 Dec 5;20(12):e0336356. doi: 10.1371/journal.pone.0336356 (PMC12680182; doi:10.1371/journal.pone.0336356)
Supplement: S5 Table — (DOCX) [file pone.0336356.s012.docx]

**S5 Table. Brain regions showing significant activation in treatment > control (green frame condition) during WTP ratings**

| **Cluster Nr.** | **Hemisphere** | **Brodmann**  **Area** | **Peak** | **x** | **y** | **z** | **Peak *t* Score** | **Cluster Size (*k*)** |
| --- | --- | --- | --- | --- | --- | --- | --- | --- |
| 1 | R | BA37 | R Fusiform | 32 | -42 | -14 | 6.51 | 1139 |
|  | R | BA21 | R Medial Temporal Gyrus | 44 | -36 | 2 | 6.11 |  |
|  | R | BA22 | R Superior Temporal Gyrus | 46 | -30 | 0 | 5.20 |  |
| 2 | R | BA10 | R Anterior Prefrontal Cortex | 36 | 48 | 24 | 5.94 | 407 |
|  | R | BA9 | R Dorsal Dorsolateral Prefrontal Cortex | 32 | 36 | 24 | 3.85 |  |
| 3 | R | BA9 | R Lateral Dorsolateral Prefrontal Cortex | 46 | 24 | 22 | 5.53 | 394 |
|  | R | BA44 | R Broca’s Area & Opercular Cortex | 34 | 2 | 30 | 5.28 |  |
| 4 | L | BA37 | L Fusiform | -28 | -56 | -10 | 4.71 | 206 |
| 5 | L | BA46 | L Lateral Dorsolateral Prefrontal Cortex | -28 | 40 | 8 | 5.27 | 150 |
|  | L | BA10 | L Anterior Prefrontal Cortex | -18 | 42 | 14 | 3.77 |  |
|  | L | BA45 | Broca’s Area (Pars Triangularis) | -42 | 32 | 8 | 3.67 |  |
| 6 | R | BA18 | R Secondary Visual Cortex | 2 | -78 | -2 | 4.92 | 105 |

*Note.* Threshold *T* = 3.56, *p* _uncorrected_ (two-sided, voxel/peak level) < .001, cluster defining threshold (cluster size, in voxels) => 105 voxels, *p _FWE_* _corrected_ (cluster level) < .05, df = [1,39]. No regions showed higher activation in control than treatment and only unidirectional effects were found. Cluster size is displayed in number of voxels. The table shows additional local maxima more than 4.0 mm apart. Clusters with multiple peaks in the same brain region are only reported once. L= Left; R = Right.
