# Supplementary material for: From attributes to value: Neural correlates of a front-of-package label on food decision-making – An fMRI study
Source: PLoS One. 2025 Dec 5;20(12):e0336356. doi: 10.1371/journal.pone.0336356 (PMC12680182; doi:10.1371/journal.pone.0336356)
Supplement: S6 Table — (DOCX) [file pone.0336356.s013.docx]

**S6 Table. Brain regions showing significant activation in treatment > control (green frame condition) during healthiness ratings.**

| **Cluster Nr.** | **Hemisphere** | **Brodmann**  **Area** | **Peak** | **x** | **y** | **z** | **Peak t Score** | **Cluster Size (k)** |
| --- | --- | --- | --- | --- | --- | --- | --- | --- |
| 1 | R | - | Cerebellum | 54 | -50 | -50 | 10.31 | 36529 |
|  | R | BA39 | Angular Gyrus | 50 | -56 | 42 | 10.29 |  |
|  | L | BA39 | Angular Gyrus | -42 | -56 | 44 | 10.00 |  |
|  | R | BA21 | Medial Temporal Gyrus | 62 | -28 | -8 | 9.86 |  |
|  | R | BA7 | Visual Motor Cortex | 4 | -72 | 42 | 9.57 |  |
|  | R | BA10 | Anterior Prefrontal Cortex | 36 | 56 | 12 | 9.57 |  |
|  | L | BA39 | Angular Gyrus | -50 | -58 | 42 | 9.53 |  |
|  | R | BA9 | Dorsal Dorsolateral Prefrontal Cortex | 50 | 26 | 34 | 9.46 |  |
|  | R | BA8 | Frontal Eye Fields | 48 | 18 | 44 | 9.39 |  |
|  | L | BA40 | Supramarginal Gyrus | -40 | -48 | 42 | 9.24 |  |
| 2 | L | BA21 | Medial Temporal Gyrus | -56 | -42 | -4 | 9.7 | 1440 |
|  | L | BA38 | Temporal Pole | -58 | 4 | -24 | 6.09 |  |
|  | L | BA20 | Inferior Temporal Gyrus | -54 | -26 | -20 | 5.34 |  |
|  | L | BA39 | Angular Gyrus | -48 | -54 | 8 | 3.77 |  |
| 3 | L | BA24 | Ventral Anterior Cingulate | -12 | 32 | 18 | 4.25 | 95 |
|  | L | BA32 | Dorsal Anterior Cingulate Cortex | -8 | 38 | 8 | 4.24 |  |

*Note.* Threshold *T* = 3.56, *p* _uncorrected_ (two-sided, voxel/peak level) < .001, cluster defining threshold (cluster size, in voxels) => 95 voxels, *p _FWE_* _corrected_ (cluster level) < .05, df = [1,39]. No regions showed higher activation in control than treatment and only unidirectional effects were found. Cluster size is displayed in number of voxels. The table shows additional local maxima more than 4.0 mm apart. Clusters with multiple peaks in the same brain region are only reported once. L= Left; R = Right.
