# Supplementary material for: From attributes to value: Neural correlates of a front-of-package label on food decision-making – An fMRI study
Source: PLoS One. 2025 Dec 5;20(12):e0336356. doi: 10.1371/journal.pone.0336356 (PMC12680182; doi:10.1371/journal.pone.0336356)
Supplement: S7 Table — (DOCX) [file pone.0336356.s014.docx]

**S7 Table. Brain regions showing significant activation in treatment > control (green frame condition) during tastiness ratings.**

| **Cluster Nr.** | **Hemisphere** | **Brodmann**  **Area** | **Peak** | **x** | **y** | **z** | **Peak *t* Score** | **Cluster Size (*k*)** |
| --- | --- | --- | --- | --- | --- | --- | --- | --- |
| 1 | R | BA21 | Medial Temporal Gyrus | 66 | -38 | 0 | 6.41 | 4760 |
|  | R | BA39 | Angular Gyrus | 36 | -54 | 44 | 6.36 |  |
|  | L | BA7 | Visual Motor Cortex | -4 | -74 | 40 | 5.85 |  |
|  | R | BA38 | Temporal Pole | 56 | 8 | -24 | 5.80 |  |
|  | R | BA40 | Supramarginal Gyrus | 54 | -50 | 48 | 5.76 |  |
|  | R | BA8 | Visual Motor Cortex | 10 | -52 | 50 | 5.56 |  |
|  | R | BA22 | Superior Temporal Gyrus | 48 | -44 | 16 | 5.21 |  |
|  | R | BA37 | Fusiform | 56 | -48 | 2 | 5.14 |  |
| 2 | R | BA10 | Anterior Prefrontal Cortex | 22 | 62 | 18 | 7.22 | 3789 |
|  | R | BA9 | Dorsal Dorsolateral Prefrontal Cortex | 52 | 26 | 20 | 6.37 |  |
|  | R | BA44 | Broca’s Area & Opercular Cortex | 42 | 12 | 30 | 5.45 |  |
|  | R | BA46 | Lateral Dorsal Prefrontal Cortex | 42 | 40 | 2 | 5.44 |  |
|  | R | BA47 | Pars Orbitalis | 50 | 42 | -10 | 5.29 |  |
| 3 | L | BA10 | Anterior Prefrontal Cortex | -20 | 58 | 20 | 5.83 | 2307 |
|  | L | BA46 | Lateral Dorsolateral Prefrontal Cortex | -36 | 36 | 16 | 5.61 |  |
|  | L | BA9 | Dorsal Dorsolateral Prefrontal Cortex | -38 | 26 | 22 | 5.49 |  |
|  | L | BA45 | Broca’s Area (Pars Triangularis) | -44 | 20 | 8 | 5.11 |  |
|  | L | BA13 | Insula | -40 | 14 | 6 | 4.94 |  |
| 4 |  | BA40 | Supramarginal Gyrus | -44 | -44 | 40 | 5.36 | 744 |
|  | L | BA30 | Angular Gyrus | -52 | -46 | 46 | 5.23 |  |
|  | L | BA7 | Visual Motor Cortex | -36 | -62 | 54 | 4.54 |  |
| **Cluster Nr.** | **Hemisphere** | **Brodmann**  **Area** | **Peak** | **x** | **y** | **z** | **Peak *t* Score** | **Cluster Size (*k*)** |
|  | L | BA39 | Angular Gyrus | -50 | -56 | 44 | 4.43 |  |
| 5 | R | BA23 | Ventral Posterior Cingulate Cortex | 4 | -42 | 26 | 5.48 | 468 |
|  | R | BA31 | Dorsal Posterior Cingulate Cortex | 4 | -28 | 48 | 4.78 |  |
|  | L | BA31 | Dorsal Posterior Cingulate Cortex | -6 | -24 | 38 | 4.32 |  |
| 6 | R | BA9 | Dorsal Dorsolateral Prefrontal Cortex | 2 | 46 | 46 | 5.06 | 409 |
|  | R | BA8 | Frontal Eye Fields | 6 | 28 | 56 | 5.05 |  |
|  | R | BA6 | Premotor Cortex+ Supplementary Motor | 6 | 20 | 60 | 4.27 |  |
|  | L | BA8 | Frontal Eye Fields | -2 | 40 | 48 | 4.22 |  |
| 7 | L | BA21 | Medial Temporal Gyrus | -64 | -46 | -8 | 5.25 | 337 |
|  | L | BA37 | Fusiform | -62 | -50 | -2 | 4.74 |  |
| 8 | R | BA37 | Fusiform | 32 | -40 | -16 | 4.82 | 149 |
|  | R | BA36 | Parahippocampus | 36 | -34 | -16 | 4.61 |  |
| 9 | R | BA17 | Primary Visual Cortex | 2 | -82 | 0 | 5.6 | 138 |
| 10 | L | - | Cerebellum | -12 | -84 | -28 | 5.33 | 104 |

*Note.* Threshold *T* = 3.56, *p* _uncorrected_ (two-sided, voxel/peak level) < .001, cluster defining threshold (cluster size, in voxels) => 104 voxels, *p _FWE_* _corrected_ (cluster level) < .05, df = [1,39]. No regions showed higher activation in control than treatment and only unidirectional effects were found. Cluster size is displayed in number of voxels. The table shows additional local maxima more than 4.0 mm apart. Clusters with multiple peaks in the same brain region are only reported once. L= Left; R = Right.
