# Supplementary material for: From attributes to value: Neural correlates of a front-of-package label on food decision-making – An fMRI study
Source: PLoS One. 2025 Dec 5;20(12):e0336356. doi: 10.1371/journal.pone.0336356 (PMC12680182; doi:10.1371/journal.pone.0336356)
Supplement: S8 Table — (DOCX) [file pone.0336356.s015.docx]

**S8 Table.** **Brain regions showing significant activation in treatment > control (yellow frame condition) during WTP ratings.**

| **Cluster Nr.** | **Hemisphere** | **Brodmann Area** | **Peak** | **x** | **Y** | **z** | **Peak t Score** | **Cluster Size (k)** |
| --- | --- | --- | --- | --- | --- | --- | --- | --- |
| 1 | R | BA37 | R Fusiform | 30 | -70 | -4 | 6.32 | 1127 |
|  | R | BA18 | R Visual Association Cortex | 24 | -58 | -8 | 6.01 |  |
|  | R | BA17 | R Primary Visual Cortex | 2 | -82 | 0 | 5.40 |  |
| 2 | R | BA10 | R Anterior Prefrontal Cortex | 22 | 62 | 18 | 5.19 | 342 |
|  | R | BA9 | R Dorsal Dorsolateral Prefrontal Cortex | 20 | 50 | 26 | 3.80 |  |
| 3 | L | BA37 | L Fusiform | -36 | -44 | -16 | 5.03 | 230 |
| 4 | R | BA21 | R Medial Temporal Gyrus | 44 | -34 | 0 | 5.01 | 218 |
|  | R | BA37 | R Fusiform | 50 | -48 | 4 | 4.53 |  |
| 5 | R | BA6 | R Premotor Cortex+ Supplementary Motor Area | 42 | 6 | 30 | 5.25 | 198 |
|  | R | BA44 | R Broca’s Area & Opercular Cortex | 30 | 8 | 30 | 4.12 |  |
| 6 | R | BA7 | R Visual Motor | 28 | -64 | 40 | 4.86 | 189 |
| 7 | L | BA32 | L Dorsolateral Anterior Cingulate Cortex | -16 | 48 | 16 | 5.08 | 166 |
|  | L | BA10 | L Anterior Prefrontal Cortex | -22 | 58 | 16 | 4.24 |  |
| 8 | L | BA32 | L Dorsolateral Anterior Cingulate Cortex | -16 | 10 | 36 | 5.01 | 157 |
| 9 | R | BA38 | R Temporal Pole | 56 | 8 | -24 | 5.74 | 143 |
| 10 | L | BA9 | L Dorsal Dorsolateral Prefrontal Cortex | -38 | 26 | 22 | 5.01 | 136 |
|  | L | BA46 | L Lateral Dorsolateral Prefrontal Cortex | -36 | 32 | 16 | 4.45 |  |

*Note.* Threshold *T* = 3.56, *p* _uncorrected_ (two-sided, voxel/peak level) < .001, cluster defining threshold (cluster size, in voxels) => 114 voxels, *p _FWE_* _corrected_ (cluster level) < .05, df = [1,39]. No regions showed higher activation in control than treatment and only unidirectional effects were found. Cluster size is displayed in number of voxels. The table shows additional local maxima more than 4.0 mm apart. Clusters with multiple peaks in the same brain region are only reported once. L= Left; R = Right.
