# Supplementary material for: From attributes to value: Neural correlates of a front-of-package label on food decision-making – An fMRI study
Source: PLoS One. 2025 Dec 5;20(12):e0336356. doi: 10.1371/journal.pone.0336356 (PMC12680182; doi:10.1371/journal.pone.0336356)
Supplement: S9 Table — (DOCX) [file pone.0336356.s016.docx]

**S9 Table.** **Brain regions showing significant activation in treatment > control (yellow frame condition) during healthiness ratings.**

| **Cluster Nr.** | **Hemisphere** | **Brodmann Area** | **Peak** | **x** | **y** | **z** | **Peak t Score** | **Cluster Size (k)** |
| --- | --- | --- | --- | --- | --- | --- | --- | --- |
| 1 | R | BA8 | Frontal Eye Fields | 38 | 14 | 34 | 9.92 | 35319 |
|  | R | BA21 | Medial Temporal Gyrus | 66 | -40 | -2 | 9.18 |  |
|  | L | BA21 | Medial Temporal Gyrus | -58 | -42 | -2 | 9.10 |  |
|  | L | BA7 | Visual Motor | -34 | -48 | 48 | 8.56 |  |
|  | R | BA37 | Fusiform | 60 | -54 | -10 | 6.27 |  |
|  | L | BA40 | Supramarginal Gyrus | -38 | -48 | 42 | 7.96 |  |
|  | L | BA39 | Angular Gyrus | -52 | -46 | 46 | 7.88 |  |
|  | R | BA39 | Angular Gyrus | 34 | -50 | 42 | 7.77 |  |
|  | R | BA6 | Premotor Cortex+ Supplementary Motor |  |  |  |  |  |
| 2 | L | - | Cerebellum | -28 | -70 | -30 | 5.15 | 426 |
| 3 | L | BA47 | Pars Orbitalis | -36 | 22 | -12 | 4.98 | 208 |
|  | L | BA38 | Temporal Pole | -44 | 16 | -20 | 4.22 |  |
| 4 | L | - | Cerebellum | -8 | -48 | -4 | 4.67 | 193 |
| 5 | R | BA30 | Agranular Retrolimbic Cortex | 4 | -44 | 8 | 6.46 | 155 |
|  | R | BA36 | Parahippocamus | 6 | -40 | -2 | 4.36 |  |
| 6 | R | - | Thalamus | 12 | -4 | 4 | 5.36 | 124 |
| 7 | R | - | Cerebellum | 12 | -52 | -46 | 5.23 | 93 |
| 8 | L | - | Brainstem | -8 | -26 | -14 | 4.91 | 93 |
| 9 | L | BA38 | Temporal Pole | -58 | 2 | -26 | 6.15 | 84 |

*Note.* Threshold *T* = 3.56, *p* _uncorrected_ (two-sided, voxel/peak level) < .001, cluster defining threshold (cluster size, in voxels) => 84 voxels, *p _FWE_* _corrected_ (cluster level) < .05, df = [1,39]. No regions showed higher activation in control than treatment and only unidirectional effects were found. Cluster size is displayed in number of voxels. The table shows additional local maxima more than 4.0 mm apart. Clusters with multiple peaks in the same brain region are only reported once. L= Left; R = Right.
