# Supplementary material for: From attributes to value: Neural correlates of a front-of-package label on food decision-making – An fMRI study
Source: PLoS One. 2025 Dec 5;20(12):e0336356. doi: 10.1371/journal.pone.0336356 (PMC12680182; doi:10.1371/journal.pone.0336356)
Supplement: S10 Table — (DOCX) [file pone.0336356.s017.docx]

**S10 Table.** **Brain regions showing significant activation in treatment > control (yellow frame condition) during tastiness ratings.**

| **Cluster Nr.** | **Hemisphere** | **BrodmannArea** | **Peak** | **x** | **y** | **z** | **Peak *t* Score** | **Cluster Size (*k*)** |
| --- | --- | --- | --- | --- | --- | --- | --- | --- |
| 1 | R | BA21 | Medial Temporal Gyrus | 62 | -38 | -2 | 9.71 | 12960 |
|  | L | BA23 | Ventral Posterior Cingulate | -2 | -42 | 24 | 7.43 |  |
|  | R | BA40 | Supramarginal Gyrus | 50 | -40 | 42 | 7.43 |  |
|  | R | BA38 | Temporal Pole | 56 | 8 | -24 | 7.18 |  |
|  | L | BA7 | Visual Motor Cortex | -8 | -74 | 44 | 7.14 |  |
|  | L | BA39 | Angular Gyrus | -50 | -48 | 40 | 6.97 |  |
|  | L | BA40 | Supramarginal Gyrus | -52 | -42 | 50 | 6.8 |  |
|  | R | BA39 | Angular Gyrus | 30 | -66 | 46 | 6.85 |  |
|  | R | BA37 | Fusiform | 58 | -52 | -2 | 6.72 |  |
| 2 | R | BA10 | Anterior Prefrontal Cortex | 40 | 50 | 22 | 7.91 | 12271 |
|  | L | BA10 | Anterior Prefrontal Cortex | -34 | 56 | 12 | 7.06 |  |
|  | R | BA8 | Frontal Eye Fields | 46 | 16 | 42 | 6.91 |  |
|  | R | BA44 | Broca’s Area & Opercular Cortex | 42 | 14 | 30 | 6.86 |  |
|  | L | BA45 | Broca’s Area (Pars Triangularis) | -30 | 28 | 12 | 6.73 |  |
|  | L | BA9 | Dorsal Dorsolateral Prefrontal Cortex | -18 | 46 | 22 | 6.33 |  |
|  | R | BA47 | Pars Orbitalis | 50 | 40 | -8 | 6.24 |  |
| 3 | R | BA18 | Secondary Visual Cortex | 2 | -76 | -2 | 6.83 | 1521 |
|  | L | - | Cerebellum | -14 | -84 | -28 | 6.68 |  |
|  | L | BA18 | Secondary Visual Cortex | -2 | -80 | -4 |  |  |
|  | R | - | Cerebellum | 4 | -92 | -10 | 5.72 |  |
| 4 | L | BA21 | Medial Temporal Gyrus | -56 | -40 | -4 | 6.86 | 1059 |
|  | L | BA37 | Fusiform | -58 | -52 | -2 | 6.64 |  |
| **Cluster Nr.** | **Hemisphere** | **Brodmann**  **Area** | **Peak** | **x** | **y** | **z** | **Peak *t* Score** | **Cluster Size (*k*)** |
|  | L | BA20 | Inferior Temporal Gyrus | -56 | -26 | -20 | 4.43 |  |
|  | L | BA22 | Superior Temporal Gyrus | -64 | -42 | 8 | 3.98 |  |
| 5 | R | BA36 | Parahippocampa Gyrus | 30 | -32 | -14 | 4.77 | 178 |
|  | R | BA37 | Fusiform | 32 | -40 | -14 | 4.73 |  |
|  | R | BA19 | Visual Association Cortex | 24 | -52 | -8 | 4.66 |  |
|  | R | BA36 | Parahippocampus | 36 | -32 | -18 | 3.87 |  |
| 6 | R | - | Cerebellum | 34 | -74 | -32 | 4.35 | 105 |

*Note.* Threshold *T* = 3.56, *p* _uncorrected_ (two-sided, voxel/peak level) < .001, cluster defining threshold (cluster size, in voxels) => 105 voxels, *p _FWE_* _corrected_ (cluster level) < .05, df = [1,39]. No regions showed higher activation in control than treatment and only unidirectional effects were found. Cluster size is displayed in number of voxels. The table shows additional local maxima more than 4.0 mm apart. Clusters with multiple peaks in the same brain region are only reported once. L= Left; R = Right.
