# Supplementary material for: From attributes to value: Neural correlates of a front-of-package label on food decision-making – An fMRI study
Source: PLoS One. 2025 Dec 5;20(12):e0336356. doi: 10.1371/journal.pone.0336356 (PMC12680182; doi:10.1371/journal.pone.0336356)
Supplement: S11 Table — (DOCX) [file pone.0336356.s018.docx]

**S11 Table.** **Brain regions showing significant activation in treatment > control (red frame condition) during WTP ratings.**

| **Cluster Nr.** | **Hemisphere** | **Brodmann**  **Area** | **Peak** | **x** | **y** | **z** | **Peak t Score** | **Cluster Size (k)** |
| --- | --- | --- | --- | --- | --- | --- | --- | --- |
| 1 | R | BA10 | Anterior Prefrontal Cortex | 16 | 58 | 18 | 7.48 | 10622 |
|  | L | BA24 | Ventral Anterior Cingulate Cortex | -10 | 2 | 28 | 7.13 |  |
|  | R | BA8 | Frontal Eye Fields | 36 | 14 | 34 | 6.63 |  |
|  | L | BA45 | Broca’s Area (Pars Triangularis) | -28 | 28 | 12 | 6.52 |  |
|  | L | BA10 | Anterior Prefrontal Cortex | -30 | 46 | -4 | 6.40 |  |
|  | R | BA10 | Anterior Prefrontal Cortex | 32 | 56 | 16 | 6.24 |  |
|  | R | BA44 | Broca’s Area & Opercular Cortex | 44 | 8 | 28 | 6.09 |  |
|  | R | BA6 | Premotor Cortex+ Supplementary Motor Area | 36 | 2 | 54 | 6.08 |  |
|  | R | BA45 | Broca’s Area (Pars Triangularis) | 36 | 32 | 8 | 5.92 |  |
| 2 | R | BA21 | Medial Temporal Gyrus | 60 | -44 | 2 | 7.1 | 3846 |
|  | R | BA37 | Fusiform | 32 | -56 | -12 | 6.97 |  |
|  | R | BA38 | Temporal Pole | 56 | 8 | -22 | 6.76 |  |
|  | R | BA36 | Parahippocampus | 36 | -34 | -16 | 6.46 |  |
|  | R | BA19 | Visual Association | 26 | -54 | -6 | 5.60 |  |
| 3 | L | BA5 | Sensory Association Cortex | -20 | -32 | 40 | 5.92 | 1492 |
|  | L | BA31 | Dorsal Posterior Cingulate Cortex | -2 | -50 | 46 | 5.69 |  |
|  | L | BA23 | Ventral Posterior Cingulate Cortex | -2 | -42 | 26 | 5.13 |  |
|  | R | BA23 | Ventral Posterior Cingulate Cortex | 14 | -52 | 30 | 5.02 |  |
|  | R | BA31 | Dorsal Posterior Cingulate Cortex | 6 | -48 | 46 | 4.94 |  |
| 4 | R | BA39 | Angular Gyrus | 30 | -66 | 42 | 5.38 | 1253 |
|  | R | BA7 | Visual Motor Cortex | 34 | -52 | 46 | 4.25 |  |
| **Cluster Nr.** | **Hemisphere** | **Brodmann**  **Area** | **Peak** | **x** | **y** | **z** | **Peak t Score** | **Cluster Size (k)** |
|  | R | BA19 | Visual Association Cortex | 32 | -88 | 18 | 4.81 |  |
|  | R | BA18 | Secondary Visual Cortex | 24 | -84 | 20 | 4.32 |  |
| 5 | L | BA21 | Medial Temporal Gyrus | -56 | -44 | -2 | 7.00 | 967 |
|  | L | BA37 | Fusiform | -52 | -40 | -12 | 4.83 |  |
|  | L | BA20 | Inferior Temporal Gyrus | -42 | -32 | -16 | 4.72 |  |
| 6 | R | - | Cerebellum | 14 | -76 | -32 | 7.06 | 916 |
|  | L | - | Cerebellum | -10 | -78 | -36 | 6.32 |  |
| 7 | L | BA37 | Fusiform | -34 | -42 | -14 | 5.22 | 609 |
|  | L | BA36 | Parahippocamus | -30 | -28 | -18 | 4.89 |  |
|  | L | BA39 | Angular Gyrus | -42 | -58 | -14 | 4.58 |  |
|  | L | BA19 | Visual Association Cortex | -32 | -66 | -14 | 4.55 |  |
| 8 | L | BA21 | Medial Temporal Gyrus | -60 | 0 | -18 | 5.23 | 478 |
|  | L | BA38 | Temporal Pole | -58 | 4 | -24 | 5.08 |  |
|  | L | BA47 | Pars Orbitalis | -32 | 20 | -22 | 4.87 |  |
| 9 | R | BA7 | Visual Motor Cortex | 2 | -72 | 40 | 5.29 | 407 |
|  | L | BA7 | Visual Motor Cortex | -8 | -74 | 44 | 5.06 |  |
|  | L | BA31 | Dorsal Posterior Cingulate Cortex | -2 | -60 | 38 | 4.33 |  |
| 10 |  | BA7 | Visual Motor Cortex | -34 | -60 | 54 | 4.91 | 377 |
| 11 | L | BA8 | Frontal Eye Fields | -46 | 16 | 40 | 4.26 | 170 |
|  | L | BA6 | Premotor Cortex+ Supplementary Motor | -48 | 10 | 46 | 4.17 |  |
| 12 | L | BA21 | Medial Temporal Gyrus | -54 | -48 | 10 | 4.90 | 137 |
| 13 | R | BA17 | Primary Visual Gyrus | 2 | -82 | 0 | 5.22 | 120 |
| 14 | R | - | Thalamus | 8 | -4 | 2 | 5.77 | 92 |
|  | L |  | Thalamus | -4 | -10 | -10 | 4.45 |  |

*Note.* Threshold *T* = 3.56, *p* _uncorrected_ (two-sided, voxel/peak level) < .001, cluster defining threshold (cluster size, in voxels) => 91 voxels, *p _FWE_* _corrected_ (cluster level) < .05, df = [1,39]. No regions showed higher activation in control than treatment and only unidirectional effects were found. Cluster size is displayed in number of voxels. The table shows additional local maxima more than 4.0 mm apart. Clusters with multiple peaks in the same brain region are only reported once. L= Left; R = Right.
