# Supplementary material for: From attributes to value: Neural correlates of a front-of-package label on food decision-making – An fMRI study
Source: PLoS One. 2025 Dec 5;20(12):e0336356. doi: 10.1371/journal.pone.0336356 (PMC12680182; doi:10.1371/journal.pone.0336356)
Supplement: S12 Table — (DOCX) [file pone.0336356.s019.docx]

**S12 Table.** **Brain regions showing significant activation in treatment > control (red frame condition) during healthiness ratings.**

| **Cluster Nr.** | **Hemisphere** | **Brodmann**  **Area** | **Peak** | **x** | **y** | **z** | **Peak *t* Score** | **Cluster Size (*k*)** |
| --- | --- | --- | --- | --- | --- | --- | --- | --- |
| 1 | R | BA21 | Medial Temporal Gyrus | 64 | -40 | -2 | 10.61 | 31625 |
|  | L | BA21 | Medial Temporal Gyrus | -56 | -44 | -2 | 10.49 |  |
|  | R | BA39 | Angular Gyrus | 48 | -56 | 42 | 10.14 |  |
|  | L | BA39 | Angular Gyrus | -44 | -54 | 40 | 9.79 |  |
|  | R | BA8 | Frontal Eye Fields | 42 | 10 | 46 | 9.61 |  |
|  | R | BA10 | Anterior Prefrontal Cortex | 16 | 62 | 18 | 9.39 |  |
| 2 | L | - | Cerebellum | -12 | -82 | -30 | 5.95 | 398 |
| 3 | R | - | Cerebellum | 16 | -78 | -28 | 4.91 | 142 |
| 4 | R | BA18 | Secondary Visual Cortex | 4 | -90 | -10 | 4.66 | 116 |
|  | R | BA17 | Primary Visual Cortex | 2 | -82 | 0 | 4.14 |  |
| 5 | L | - | Cerebellum | -8 | -52 | -46 | 5.84 | 83 |

*Note.* Threshold *T* = 3.56, *p* _uncorrected_ (two-sided, voxel/peak level) < .001, cluster defining threshold (cluster size, in voxels) => 83 voxels, *p _FWE_* _corrected_ (cluster level) < .05, df = [1,39]. No regions showed higher activation in control than treatment and only unidirectional effects were found. Cluster size is displayed in number of voxels. The table shows additional local maxima more than 4.0 mm apart. Clusters with multiple peaks in the same brain region are only reported once. L= Left; R = Right.
