# Supplementary material for: From attributes to value: Neural correlates of a front-of-package label on food decision-making – An fMRI study
Source: PLoS One. 2025 Dec 5;20(12):e0336356. doi: 10.1371/journal.pone.0336356 (PMC12680182; doi:10.1371/journal.pone.0336356)
Supplement: S13 Table — (DOCX) [file pone.0336356.s020.docx]

**S13 Table.** **Brain regions showing significant activation in treatment > control (red frame condition) during tastiness ratings**

| **Cluster Nr.** | **Hemisphere** | **Brodmann**  **Area** | **Peak** | **x** | **y** | **z** | **Peak *t* Score** | **Cluster Size (*k*)** |
| --- | --- | --- | --- | --- | --- | --- | --- | --- |
| 1 | R | BA21 | Medial Temporal Gyrus | 62 | -26 | -8 | 10.84 | 38665 |
|  | R | BA8 | Frontal Eye Fields | 0 | 38 | 48 | 8.99 |  |
|  | L | BA39 | Angular Gyrus | -50 | -48 | 42 | 8.30 |  |
|  | R | BA10 | Anterior Prefrontal Cortex | 14 | 58 | 16 | 8.06 |  |
|  | R | BA7 | Visual Motor Cortex | 10 | -52 | 50 | 8.01 |  |
|  | L | BA10 | Anterior Prefrontal Cortex | -38 | 54 | 8 | 7.98 |  |
|  | R | BA21 | Medial Temporal Gyrus | 68 | -40 | -2 | 7.98 |  |
|  | L | BA21 | Medial Temporal Gyrus | -60 | -44 | -2 | 7.90 |  |
|  | R | BA40 | Supramarginal Gyrus | 54 | -40 | 48 | 7.66 |  |
|  | R | BA9 | Dorsal Dorsolateral Prefrontal Cortex | 38 | 32 | 32 | 7.26 |  |
| 2 | L | - | Cerebellum | -12 | -84 | -28 | 7.87 | 1458 |
|  | R | BA18 | Secondary Visual Cortex | 4 | -86 | -8 | 6.49 |  |
| 3 | L | BA37 | Fusiform | -36 | -46 | -10 | 5.40 | 309 |
|  | L | BA36 | Parahippocampus | -20 | -26 | -12 | 4.19 |  |
|  | L | - | Hippocampus | -34 | -26 | -12 | 3.60 |  |
| 4 | L | - | Cerebellum | -8 | -48 | -6 | 4.81 | 130 |
| 5 | L | - | Hippocampus | -36 | -18 | -18 | 5.20 | 118 |
| 6 | R | - | Thalamus | 10 | -4 | 0 | 5.11 | 116 |
|  | R | - | Amygdala | 16 | -4 | -20 | 4.67 |  |

*Note.* Threshold *T* = 3.56, *p* _uncorrected_ (two-sided, voxel/peak level) < .001, cluster defining threshold (cluster size, in voxels) => 116 voxels, *p _FWE_* _corrected_ (cluster level) < .05, df = [1,39]. No regions showed higher activation in control than treatment and only unidirectional effects were found. Cluster size is displayed in number of voxels. The table shows additional local maxima more than 4.0 mm apart. Clusters with multiple peaks in the same brain region are only reported once. L= Left; R = Right.
