# Supplementary material for: From attributes to value: Neural correlates of a front-of-package label on food decision-making – An fMRI study
Source: PLoS One. 2025 Dec 5;20(12):e0336356. doi: 10.1371/journal.pone.0336356 (PMC12680182; doi:10.1371/journal.pone.0336356)
Supplement: Attachment — Submitted filename: Response to Reviewers.docx [file pone.0336356.s022.docx]

From,

Negin Javaheri

Department of Neuropsychology and Behavioral Neurobiology,

University of Bremen

To,

Dr. Ogawa

**Subject:** Rebuttal Letter for Manuscript PONE-D-25-19520

From attributes to value: neural correlates of a front-of-package label on food decision-making – an fMRI study

PLOS ONE

Dear Dr. Ogawa,

We sincerely appreciate the invitation to revise and resubmit our Manuscript PONE-D-25-19520entitled " From attributes to value: neural correlates of a front-of-package label on food decision-making – an fMRI study" to PLoS One. We are grateful for the valuable comments provided by you, as well as both of the reviewers that helped us to clarify and improve the quality of our work.

Below, we will provide a detailed response to each of the comments. For the sake of clarity, the journal requirements and the reviewers’ comments are included in bold, followed by our responses in plain text. All changes made to the manuscript are attached as ‘Revised_Manuscript_PONE-D-25-19520_with_Track_Changes’, further we attach the manuscript without track mode as ‘Revised_Manuscript_ PONE-D-25-19520’. Additionally, we revised the *Discussion* section (see page 23, line 515 ff.). Please note that the page and line references are based on the revised manuscript with track changes.

Thank you for your time.

Best Regards,

Negin Javaheri

**Journal Requirements:**

1. **When submitting your revision, we need you to address these additional requirements. Please ensure that your manuscript meets PLOS ONE's style requirements, including those for file naming.**

We apologize for the inconsistencies in the submission. We have revised the manuscript according to the requirements.

2. **Thank you for stating the following financial disclosure: This work was funded by Deutsche Forschungsgemeinschaft (DFG, German Research Foundation)-GRK2739/1-Project Nr. 447089431-Research Training Group: KD2 School-Designing Adaptive Systems for Economic Decisions.** **Please state what role the funders took in the study. If the funders had no role, please state: "The funders had no role in study design, data collection and analysis, decision to publish, or preparation of the manuscript." If this statement is not correct you must amend it as needed. Please include this amended Role of Funder statement in your cover letter; we will change the online submission form on your behalf.**

We thank for the additional indication. We have extended the sentence and deleted in the revised manuscript (see page 34, line 849 ff.)

3. **Please note that your Data Availability Statement is currently missing the repository name and/or the DOI/accession number of each dataset and a direct link to access each database. If your manuscript is accepted for publication, you will be asked to provide these details on a very short timeline. We therefore suggest that you provide this information now, though we will not hold up the peer review process if you are unable.**

As we are commited to principles of open science, we will be making the data available under OSF. The DOI will be given when the manuscript will be accepted. Currently, we are preparing the data for publication, e.g. defacing fMRI data for anonymization.

4. **When completing the data availability statement of the submission form, you indicated that you will make your data available on acceptance. We strongly recommend all authors decide on a data sharing plan before acceptance, as the process can be lengthy and hold up publication timelines. Please note that, though access restrictions are acceptable now, your entire data will need to be made freely accessible if your manuscript is accepted for publication. This policy applies to all data except where public deposition would breach compliance with the protocol approved by your research ethics board. If you are unable to adhere to our open data policy, please kindly revise your statement to explain your reasoning and we will seek the editor's input on an exemption. Please be assured that, once you have provided your new statement, the assessment of your exemption will not hold up the peer review process.**

We appreciate this suggestion and have made a data sharing plan. We will be uploading the MRI data in OSF, once we finished the defacing to ensure anonymity. Additionally, we will upload all analysis codes, and all behavioral data set both for studies referred to in the present manuscript as well as piloting and other data mentioned here.

**Reviewer#1**

1. **Fixed order of conditions: The control trials always preceded the treatment trials. This fixed sequence could be a critical confound: differences in behavior and neural activity may reflect order effects rather than the manipulation itself. Please discuss this issue.**

We thank the reviewer for highlighting this important concern, which is also shared by the reviewer 2 (point 7). We fully acknowledge that presenting the control trials before the treatment trials may have introduced order effects, potentially confounding behavioral and neural differences. We have now addressed this point explicitly in the *Limitations* section. Our rationale for using a fixed order was to avoid potential carryover effects from the treatment condition onto the control trials. Presenting the treatment condition first might have biased participants' baseline responses, undermining the interpretability of the control condition. Nevertheless, we agree that the fixed sequence limits the ability to dissociate treatment effects from possible order effects. We now discuss this possible confound in the Limitations section (see page 29, line 720 ff.).

2. **Visual disparity between conditions: The treatment condition included a colored frame around each food image, whereas the control condition did not. Consequently, any behavioral or neural differences might stem from the mere presence of salient color cues rather than their Nutri-Score meaning. Please consider addressing this alternative explanation.**

We acknowledge the reviewer’s concern that the color-coded frame used in the treatment condition may have served as a salient visual cue rather than being interpreted as meaningful information on healthiness of food components. This issue was also raised by Reviewer 2 (see point 3) has been discussed within our research team, and was subject on various analyses and pilot studies not mentioned in the present manuscript. We would like to stretch the various reasons behind using a color-coded frame and not just the Nutri-Score label including a colored scheme as well. From the neuroeconomics literature, we know that attention strongly shapes decision-making. Fehr & Rangel (2011), include attention as one of the main factors that control decisions. Further, from the work by Reeck and colleagues (2017), we know that manipulation of attention can alter overall choice. With respect to experimental reasons The here used color-coded frame allowed us to control for how much attention participants gave to the treatment, as on a visual level there was no escape from the frame. If a label was used, then the frame could have been ignored, which would also mean that our treatment might have not worked. In the following, we show in two different analyses that corroborates our view that the frame was seen as more than a color cue and participants perceived it as a frame containing information of the healthiness of the product’s nutritional components.

As a first measure to assess this issue whether the color-coded frame was interpreted as nutritional information rather than merely a salient visual cue, we analyzed participants' responses from a post-experiment questionnaire. All participants were asked to re-rate the products in a post-hoc questionnaire, answering the question how much they are willing to pay for the product(“ How much are you willing to pay for the product”), and how much they are willing to pay for the product given their real Nutri-Score (“ How much are you willing to pay for the product (Nutri-Score X)”) on a continuous scale. We than compared the average WTP of participants in the fMRI and in the post-hoc questionnaire to investigate whether average responses differed between the MRI setting and in the post-hoc questionnaire. A repeated-measure ANOVA was conducted using average responses of participants per setting, treatment condition, and color-coded frame. A Shapiro–Wilk test indicated that all variables were normally distributed (all p-values > 0.1), and no outliers were identified. The results of an ANOVA showed a significant main effect of setting (*F* (1, 39) = 8.84, *p* = .005, *η²* = .040), main effect of treatment (*F* (1, 39) = 28.57, *p* < .001, *η²* = .008), and main effect of the color-code (*F* (2, 78) = 56.84, *p* < .001, *η²* = .094). The finding was interpreted in a way that WTP differed between settings, between treatment, and color-codes, with a significant interaction effect between setting and treatment (*F* (1, 39) = 6.63, *p* = .014, *η²* = .002), as well as the interaction between treatment and color-coded frame (*F* (2, 78) = 52.13, *p* < .001, *η²* = .019). Further, the three-way interaction between setting, treatment condition, and color-coded frame became also significant (*F* (2, 78) = 7.75, *p* = .001, *η²* = .002), indicating that the combined influence of setting and treatment condition varied across color-coded frames. In addition, post-hoc paired *t*-tests were conducted between the settings within each color-coded frame and treatment condition with Bonferroni correction . For average WTP per setting, treatment conditions and color-coded frame conditions, see the following Figure 1.

Figure 1. Average WTP per Setting, Treatment and Color-Coded Frame Condition

In both the control condition as well as in the treatment condition, average WTP was significantly higher in the post-hoc-questionnaire setting (*M_post-hoc-control_* = 1.09, *SD_post-hoc-control_* = 0.31; *M_post-hoc-treatment_* = 1.06, *SD_post-hoc-treatment_* = 0.37 ) compared to the MRI setting (*M_MRI-control_* = 0.99, *SD_MRI-control_* = 0.32; *p_Bonf_* < .05; *M_MRI-treatment_* = 0.90, *SD_MRI-treatment_* = 0.36; *p_Bonf._* < .001). For the green color-coded frame condition, a significant difference in WTP between settings emerged both in the control group (*p_Bonf.-_* < .05) and in the treatment group (*p_Bonf._* < .001). Participants in the post-hoc-questionnaire condition reported higher WTP values (*M_post-hoc-treatment-green_* = 1.28, *SD_post-hoc-treatment-green_* = 0.28; *M_post-hoc-control-green_* = 1.16, *SD_post-hoc-control-green_* = 0.26) compared to those in the MRI condition (*M_MRI-treatment -green_*= 1.02, *SD_MRI-treatment-green_* = 0.35; *M_MRI-control-green_* = 1.03, *SD_MRI-control-green_* = 0.33). For the yellow color-coded frame, no significant difference between settings were found (*p_Bonf._*= .29 for control condition; *p_Bonf._*= .21 for treatment condition). There was a non-significant increase in WTP in the post-hoc-questionnaire setting (*M_post-hoc-treatment-yellow_* = 1.04, *SD_post-hoc-treatment-yellow_* = 0.32, *M_post-hoc-control-yellow_* = 1.10, *SD_post-hoc-control-yellow_* = 0.30) relative to the MRI setting (*M_MRI-treatment -yellow_* = 0.95, *SD_MRI-treatment-yellow_* = 0.34; *M_MRI-control-yellow_* = 1.03*, SD_MRI-control-yellow_* = 0.33). Similarly, for the red color-coded frame no significant difference between settings was found (*p_Bonf._* = .18 for treatment condition; *p_Bonf._* = .17 for control condition). Similar to the yellow-color coded condition, average WTP values were higher in the post-hoc-questionnaire setting (*M_post-hoc-treatment-red_* = 0.85, *SD_post-hoc-treatment-red_* = 0.36; *M_post-hoc-control-red_* = 1.00, *SD_post-hoc-control-red_* = 0.35) compared to the MRI condition (*M_MRI-treatment -red_* = 0.74, *SD_MRI-treatment-red_* = 0.33*; M_MRI-control-red_* = 0.90, *SD_MRI-control-red_* = 0.30), however not significantly different from each other.

Taken together, these data indicate no significant difference in treatment effects between the yellow and red color-coded conditions. While a significant effect was observed in the green condition (in the post-hoc questionnaire analysis), we believe this might be due to participants being allowed to consume their chosen product during the post-hoc questionnaire, thereby breaking their fast. A similar significant effect of a green traffic-light label on WTP was also reported by Enax et al. (2015), where participants had not been instructed to fast prior to the experiment. Therefore, the present findings strongly suggest that the effect of the color-coded frame observed in the rating task was similar to when participants were presented with the information of the actual Nutri-Score of the products. To also provide this information, we included an additional supplementary online S4 File, as well as a short reference in the Methods section (see Experimental Design and Behavioral Data Analysis; page 10, line 216 ff.) and the Discussion section (see Limitations, page 28, 706 ff.).

Besides the post-hoc questionnaire not included in the present manuscript, we also conducted an additional laboratory study to complete a neuroforecasting approach. The results of that add-on study are subject to another publication and therefore are not included in this paper. This data give further evidence that the color-coded frame was perceived as information and not only as a salient cue. In this laboratory study, 108 participants made 126 binary choices between the products that were shown in the presented study (63 trials in the control condition and 63 trials in the treatment condition, where the Nutri-Score label was shown). Percentage of product choice was calculated for each trial of the treatment condition of the binary choice task. These percentages served as our dependent variables. For our independent variables, behavioral and neural data from the rating task of the present study (fMRI study) were used. Specifically, for each product, we extracted the average activation from predefined voxels of interest (VOIs; using medial prefrontal cortex (mPFC) and nucleus accumbens (NAcc) of the right and left hemisphere) based on the Affect-Integration-Motivation (AIM) framework by Knutson and Genevsky (2018). We predicted product choice of the laboratory study (dependent variables) with average behavioral, neural data of the rating task (independent variables) as well as the difference in the Nutri-Score using linear regressions. We estimated three sets of linear regression models for WTP (dependent variable), and compared (1) a behavioral model, (2) a neural model, and (3) a combined model. All three models included the independent variable “Difference in Nutri-Score” to capture the effect of the label.

In the WTP domain, the behavioral model including the difference in WTP ratings and the difference in Nutri-Score explained approximately 10% of the variance in choice probability (R² = .10, p = .038). Neither predictor was significant at the 5% level, although the effect of Nutri-Score approached significance (p = .068). The neural model including the differences in activity in the right and left NAcc and mPFC, and Nutri-Score, accounted for a larger proportion of explained variance (R² = .25, p = .005). Here, the difference in Nutri-Score (p = .021) and the difference in right NAcc activation (p = .005) were significant predictors, with right NAcc activity negatively associated with the probability of choosing the left product. The combined model (behavioral, neural, and Nutri-Score predictors) slightly improved explanatory power (R² = .27, p = .006). In this model, the difference in right NAcc activation remained significant (p = .003). Neither WTP difference nor Nutri-Score difference reached significance when neural predictors were included. For detailed results see Table 1.

*Table . Linear Regression Models Predicting Out-of-Sample Product Choice with FoPL from the WTP Decision Domain*

*Note. All standard errors are reported in parentheses. Model (1) represents the behavioral model, Model (2) the neural model, and Model (3) the combined model.*

Taken together, the behavioral model did not significantly predict binary out-of-sample choices. In contrast, differences in neural activation within the selected VOIs significantly predicted out-of-sample product choice. Additionally, the difference in Nutri-Score was a significant predictor in the neural model. Since all data stem from the treatment conditions of both studies, the neural data reflect the influence of the FoPL within the decision-making process. According to the AIM framework, significant predictive activity in the NAcc is typically interpreted as reflecting affective responses. However, in this case, the NAcc signal negatively predicted choice. This inverse relationship suggests that the presence of the FoPL reduced affect-driven decisions, potentially redirecting participants toward more deliberative processes. These findings are consistent with the main results of the study, which show that the color-coded frame facilitated goal-directed decisions involving self-control.

Taken together, this data additionally support the interpretation that the frame was perceived not merely as a color signal, but as meaningful information about the Nutri-Score label. This underscores the role of the frame as an information-based, cognitively engaging nudge.

As the color-coded frame in our study serves as a proxy for FoPL, we acknowledge that it does not fully capture the complexity of real-world FoPLs, which typically include additional elements such as letters, numbers, or textual information (especially in the case of the Nutri-Score). We have addressed this limitation in the *Limitations* section of the manuscript (see page 28, line 695 ff.), noting that even with full Nutri-Score labels, consumers may predominantly focus on the color scheme, potentially disregarding the accompanying letter grades. To further support the interpretation that the treatment effect was driven by the Nutri-Score information rather than mere visual salience, we included the post-hoc data analysis into the Supplementary Information (S4 File).

3. **vmPFC/striatum correlations with WTP: BOLD activity in reward-processing areas such as the ventromedial prefrontal cortex and striatum is consistently correlated with WTP. Did your data replicate this established relationship? Reporting such a validation would strengthen confidence in the fMRI findings.**

We appreciate the reviewer’s suggestion to examine the relationship between activity in the vmPFC/ventral striatum and WTP to strengthen confidence in the fMRI findings, and our data indeed replicate this relationship. We conducted a parametric modulation analysis within the original general linear model. Our data (Figure 2, Table 2) revealed significant correlation between WTP and dorsal anterior cingulate cortex (dACC; BA32) and ventromedial prefrontal cortex (vmPFC; BA11). These findings are in line with a recent meta-analysis by Newton-Fenner et al. (2023), who identified consistent WTP-related activation in the left orbitofrontal cortex (OFC; BA11) and the left vmPFC (BA10/BA32) using the Becker–DeGroot–Marschak (BDM) auction method. We report this analysis in the supplementary S6 File “Parametric modulation analysis with WTP” and introduce it in the *Discussion* section (page 23, line 526 ff.).

**Figure 2.** *Neural correlates of WTP*

The t-map is projected onto an MNI_152_2009 template. A p -threshold of < .001 (two-sided) and a FWE-cluster threshold of p<.05 (df = [1, 39]) was used. Abbreviations in the figure: dACC = dorsal anterior cingulate cortex (BA32); OFC =orbital frontal cortex (BA11). Color bar indicates t-values.

**Table 2.** *Neural correlates of WTP*

| **Cluster Nr.** | **Hemisphere** | **Brodmann**  **Area** | **Peak** | **x** | **y** | **z** | **Peak *t* Score** | **Cluster Size (*k*)** |
| --- | --- | --- | --- | --- | --- | --- | --- | --- |
| 1 | L | 24 | Ventral Anterior Cingulate Cortex | -4 | 30 | 2 | 5.86 | 645 |
|  | L | 11 | Orbitofrontal Cortex | -6 | 26 | -14 | .5.67 |  |
|  | R | 32 | Dorsal Anterior Cingulate Cortex | 10 | 32 | 4 | 5.36 |  |
|  | L | 32 | Dorsal Anterior Cingulate Cortex | -2 | 42 | 2 | 4.75 |  |
| 2 | R | / | Putamen | 30 | 12 | 2 | 5.26 | 253 |
|  | R | 13 | Insula | 38 | 16 | 2 | 4.15 |  |
| 3 | L | 40 | Supramarginal Gyrus | -52 | -44 | 54 | 5.19 | 105 |
|  | L | 7 | Visual Motor Area | -40 | -44 | 48 | 4.72 |  |
|  | L | 39 | Angular Gyrus | -42 | -46 | 40 | 4.68 |  |
| 4 | R | 46 | Dorsolateral Prefrontal Cortex (lateral part) | 42 | 44 | 8 | 5.05 | 100 |
|  | R | 9 | Dorsolateral Prefrontal Cortex (dorsal part) | 44 | 38 | 20 | 4.50 |  |

*Threshold T = 3.56, p _uncorrected_ (two-sided, voxel/peak level) < .001, cluster defining threshold (cluster size, in voxels) => 100 voxels, p _FWE corrected_ (cluster level) < .05, df = [1,39]. Only unidirectional effects were found. Cluster size is displayed in number of voxels. The table shows additional local maxima more than 4.0 mm apart. Clusters with multiple peaks in the same brain region are only reported once. L = Left; R = Right.*

4. **Sample-size justification: The manuscript does not explain how the sample size was determined. Please provide a power analysis or other rationale (e.g., prior literature, resource constraints) to justify the chosen N.**

We thank the reviewer for hinting towards this important point. We added a sample size justification in the manuscript under the section Methods (see Participants and data collection, p. 8, line 180, ff.). As the sample size justification was preregistered, we further emphasized on the preregistration in that section and reason our argumentation more clearly in the manuscript. We hope that the now modified sample size justification is acceptable and gives more clarity.

**Reviewer #2**

1. **Page 6: I am a bit surprised that you have not formulated any hypotheses, especially for the food rating task (but also for the fMRI experiment). It would be interesting to be able to compare your findings to what you would expect based on the literature.**

We thank the reviewer for pointing out that the hypotheses were not easily identifiable. While we did formulate hypotheses (see also the preregistration: Javaheri et al., 2023 (page 2)), we originally used terms such as “expect” and “anticipate,” which may have obscured their status as formal hypotheses. To improve clarity and ensure the hypotheses are more transparent to the readers, we have revised the wording throughout the manuscript (see Introduction and Discussion; page 6, line 127 ff.) and now consistently use the verb “hypothesize”. We hope that this change allows seeing the stated hypotheses more clearly. Additionally, we are referring

2. **Page 8, lines 162-164: Why are the behavioral data not included in the paper? It might be interesting to see whether what happens on a neural level is in line with what participants indicate themselves.**

We acknowledge that the inclusion of the behavioral data was not stated clear enough in the manuscript. We collected and analyzed behavioral data, and the corresponding results are included in the revised manuscript in the following sections: in the Methods section (“Behavioral data analysis”, page 12, line 273 ff.), the Results section (“Behavioral data – effects of the color-coded frame on WTP, healthiness perception, and tastiness perception; page 15, line 332 ff.), and the Discussion section (“Behavioral data” ; page 21, line 460 ff.). The conducted ANOVA was part of the preregistration of the study and thus we included it in the manuscript. We revised the manuscript to more clearly emphasize these findings and explicitly highlight their relevance to the neural data. We agree that presenting the behavioral and neural results side by side offers important context for interpreting the observed effects. Accordingly, we have clarified this connection in the revised manuscript (see Introduction, page 5, line 102 ff.).

3. **Page 9, lines 179-181: Based on the title, I initially expected the study to focus specifically on the Nutri-Score front-of-pack (FOP) label. However, the experimental design uses only three colors derived from the Nutri-Score system, without incorporating its full structure—most notably, the accompanying letter grades and the complete five-color scale. These additional components are central to the Nutri-Score’s intended function, which is to avoid overly simplistic, dichotomous thinking and to provide a more nuanced, graded evaluation of product healthfulness. From a theoretical standpoint, and in line with Cadario and Chandon’s (2020) typology, the Nutri-Score is best classified as a cognitive nudge, as it provides consumers with explicit evaluative information. In contrast, the manipulation tested in your study—color-coded product frames—would more accurately be described as an affective nudge, since it relies on intuitive and emotional responses to color cues rather than direct informational content. While the neural findings you report are potentially valuable, I believe the current framing of the paper as an investigation of the neural correlates of a front-of-pack label may be misleading. A more precise positioning would clarify that the study focuses on an affective cue inspired by elements of FOP labeling, rather than a direct examination of a complete label system such as the Nutri-Score. In my opinion, the experiment does not answer the question raised in the introduction: “How do these labels alter food perception and decision-making?”.**

We fully acknowledge the concerns raised by the reviewer. As this point encompasses three distinct issues, we would like to first address the concern regarding the study’s focus on the Nutri-Score, which may have appeared misleading. We agree that the distinction between FoPL in general and the Nutri-Score specifically was not clearly articulated in the original manuscript.

Our research question investigates how FoPL might influence decision-making, using a color-coded frame as a proxy. While this frame was derived from the real Nutri-Score of the products introduced in the experimental detup of our study, the Nutri-Score in general serves merely as one example of a health-related cue rather than the central object of study. To avoid confusion and better reflect the generalizability of our approach, we have revised the whole manuscript accordingly, clarifying the terminology and adjusting the wording throughout to more accurately distinguish between FoPL and the Nutri-Score (e.g., see page 4, line 58 ff. or page 5,line 106 ff.). Moreover, we carefully revised the manuscript to ensure that the Nutri-Score is only mentioned where directly relevant, as the central research question concerns how a FoPL alters decision-making, rather than investigating the Nutri-Score specifically. As the title itself does not include the word “Nutri-Score”, we have decided to keep the title as is.

Second, we thank the reviewer for referencing the nudge classification by Cadario and Chandon (2020), which is a paper that is also referred to in the manuscript. From a functional perspective, we agree that the original Nutri-Score label was designed to provide a more nuanced evaluation of a product’s nutritional quality by combining a five-grade letter system with a corresponding five-color scale, as also stated in the section “Limitation” ( see page 28, line 695 ff.). In our study, we instead used a simplified version, a color-coded frame derived from each product’s actual Nutri-Score. In total, we used seven food categories. Within each category, we selected three products with a “green” Nutri-Score, three with a “yellow” Nutri-Score, and three with a “red” Nutri-Score. It is important to note that not all food categories contain products that are evenly distributed across the full Nutri-Score spectrum. For example, in the category of sweet snacks, due to their inherently high sugar content, it is difficult to find products that qualify for Nutri-Score A. According to the Nutri-Score algorithm, the use of sweeteners lowers the score by one unit. Similar challenges arise in other categories, such as yogurt (due to protein content), salty snacks (due to salt content), and canned products (due to salt or sugar). While some exotic products might technically meet the criteria for certain Nutri-Scores, they are likely unfamiliar to the average participant. Based on pilot study data, we ensured that all stimuli used in the experiment were familiar to our target population.

In addition to these practical considerations, including all five Nutri-Score levels would have significantly reduced statistical power. Our current design spans nine different categories, including three decision domains. Expanding this to cover all five Nutri-Score levels would have resulted in 15 conditions, which would have increased model complexity and reduced the feasibility of the analysis and an increase in scanning time.

We understand that this approach leads to the question whether the frame was really interpreted as information about the Nutri-Score, and thus leads to the question whether the color-coded frame represents a cognitive nudge or an affective one, which was the third concern of the reviewer. A similar concern is also stated by reviewer 1 (point 2). We fully acknowledge these concerns and therefore want to thank the reviewers once again for stressing this point. We kindly ask reviewer 2 to consider our argumentation (reviewer 1, point 2) as this also holds true in the mentioned points here. In summary, we showed in two different ways (both with behavioral data of the post-hoc data, , and with an additional neuroforecasting approach), that the color-coded frame was not only seen as only a color but signaled information about the Nutri-Score derived healthiness of products. All participants were informed about how the Nutri-Score was calculated and what the frame represents. Moreover, our analyses provide evidence that the color-coded frame signals information to the participants and not only a color. We have added the behavioral analysis of the post-hoc data into a supplemental S4-File, while the neuroforecasting analaysis approach is beyond the scope of this study and will be subject of another paper. Based on the current evidence, we argue that the color-coded frame functions as a cognitive nudge rather than an affective one. This interpretation directly addresses the overarching research question of how a FoPL influences food perception and choice.

4. **Following up on the previous comment, the theoretical background should elaborate on how the chosen nudge (in your case the use of colored frames) has affected consumers’ perceptions. Currently, there is very limited information on how the Nutri-Score affects WTP, healthiness perceptions and tastiness perceptions and the focus is immediately on neural activation. As I would no longer focus on the Nutri-Score in your research, I do not suggest to include these findings in the theoretical background of the paper. However, I would recommend to include some overview of how affective nudges have impacted WTP, healthiness perceptions and tastiness perceptions in previous research.**

We appreciate the reviewers comment to deepen the theoretical background in the Introduction and we agree with the reviewer that the focus of the paper is not the Nutri-Score itself but rather a general example of FoPLs. Thus, we also elaborate the part on how FoPL affect WTP, healthiness perception, and tastiness perception and altered the sections Introduction (page 5, line 106 ff.), and Discussion (page 21, line 460 ff.) focusing more on general effects of the FoPL, where the Nutri-Score counts only as an example and on our use case. As this comment is based on the previous remark of the reviewer 2, we argue that the color-coded frame can be seen as a cognitive nudge, rather than an affective one. Therefore the changes are based on how FoPL alter food perception and food choice. We hope that these changes sufficiently address the reviewer’s concerns and clarify these relevant points.

5. **I think it is important to include the fact that you ran a pretest in the paper to select the different products (which is now explained in the S2 file). Based on this pretest, I am bit worried about the 6 options you used to measure willingness to pay, given that some products were priced higher than the maximum (i.e., €3.19). Maybe this is something to include in the limitations section of your study?**

We recognize the importance of the issue highlighted by the reviewer. We have now incorporated information about the pretest into the manuscript; specifically in the Methods section under “Food product rating task” (see page 11, line 255 ff.). To address this concern, we conducted several pilot studies and additional analyses to demonstrate that the actual market prices of the products did not influence the effect of the color-coded frame on participants’ WTP, which can be found in the following paragraphs. In summary, we show that the price does not influence the effect of the treatment manipulation. First, we identified all products with a real market price above €2.50, which resulted in a subset of 12 products out of the total 63. For each of these products, we calculated the average WTP across participants separately for the control and treatment conditions. The corresponding values can be found in the table and figure below. Notably, none of the products, regardless of condition, showed particularly high WTP values (see Figure 3 and Table 3 for an overview of the products).

**Figure 3.** *Average WTP with SD (error bars) of all participants for products priced higher than 2.50€*

**Table 3.** Overview of products priced over 2.50€ with their price, average WTP in control and treatment conditions.

|  |  | **Control** | | **Treatment** | |
| --- | --- | --- | --- | --- | --- |
| **Product** | **Price [€]** | ***M*** | ***SD*** | ***M*** | ***SD*** |
| Can Product 3 (Red) | 2.99 | 0.79 | 0.72 | 0.9 | 0.66 |
| Dips Product 1 (Green) | 2.56 | 0.91 | 0.71 | 0.91 | 0.72 |
| Dips Product 3 (Yellow) | 3.19 | 0.65 | 0.48 | 0.83 | 0.61 |
| Cereal Product 3 (Green) | 3.79 | 1.1 | 0.74 | 1.1 | 0.73 |
| Cereal Product 2 (Yellow) | 2.69 | 1.15 | 0.75 | 1.4 | 0.63 |
| Cereal Product 3 (Yellow) | 3.79 | 1.06 | 0.68 | 1.15 | 0.65 |
| Nuts Product 3 (Green) | 2.89 | 1.31 | 0.58 | 1.38 | 0.64 |
| Nuts Product 2 (Yellow) | 3.59 | 1.35 | 0.56 | 1.4 | 0.51 |
| Nuts Product 3 (Yellow) | 3.79 | 1.51 | 0.66 | 1.52 | 0.72 |
| Sweet Product 3 (Green) | 2.65 | 0.72 | 0.64 | 0.85 | 0.72 |
| Sweet Product 1 (Red) | 2.59 | 1.29 | 0.8 | 1.25 | 0.76 |
| Sweet Product 2 (Yellow) | 2.99 | 0.79 | 0.7 | 1.15 | 0.73 |

To further examine the influence of real product prices on WTP, we conducted a mixed-effects regression analysis with participants’ WTP as the dependent variable. Product price, treatment condition, and their interaction were included as fixed effects, while participant ID and product were modeled as random effects (see Table 4). The results indicate that product price is a significant predictor of WTP. However, the interaction between price and treatment condition was not significant, suggesting that product price does not moderate the treatment effect, which is the primary focus of the study. This analysis can be found now in the supplementary S2 File.

**Table 4.** Mixed-effects regressions predicting WTP from product price and treatment condition.

| **Predictors** | **Estimates** | **Std. Error** | ***CI*** | ***p*** |
| --- | --- | --- | --- | --- |
| Intercept | 0.75 | 0.09 | 0.57 – 0.93 | < .001 |
| Price | 0.08 | 0.04 | 0.01 – 0.15 | .032 |
| Treatment | 0.07 | 0.04 | -0.02 -0.16 | .113 |
| Price x Treatment | 0.00559 | 0.02 | -0.04 -0.05 | .794 |
| **Random effects** |  |  |  |  |
| *AIC* | 8881.893 |  |  |  |
| Conditional *R*^2^ | 0.295 |  |  |  |
| Marginal *R*^2^ | 0.012 |  |  |  |
| *ICC* | 0.286 |  |  |  |
| Observations | 4897 |  |  |  |
| *N*_Subjects_ | 40 |  |  |  |
| *N*_Products_ | 63 |  |  |  |

6. **Although the fMRI sample size (n = 40) is solid, the behavioral sample may be underpowered. A formal power analysis would help justify the adequacy of this sample.**

We thank the reviewer for raising this important point. To address this concern, we conducted post hoc power analyses using G*Power (version 3.1.9.7) for each of the three repeated-measures ANOVAs included in our behavioral analysis (WTP, healthiness perception, and tastiness perception).

Based on the observed partial eta-squared values, we calculated the corresponding Cohen’s f effect sizes:

- WTP (main effect of treatment): η²ₚ = 0.35 → f = 0.73
- Healthiness perception (treatment × color interaction): η²ₚ = 0.61 → f = 1.25
- Tastiness perception (main effect of treatment): η²ₚ = 0.37 → f = 0.77

For all analyses, we used the following parameters: α = 0.05, total sample size = 40, 1 group (within-subjects), 3 measurements, an estimated correlation among repeated measures of 0.5, and a nonsphericity correction ε = 0.75.

Each of the estimations indicates that the behavioral sample was sufficiently powered to detect the observed effects.

7. **Page 9, lines 184-185: Although I understand why you first showed the control trials, I would have suggested to counterbalance across participants and present the control trials first to some participants and the experimental trials first to others. You now introduce order confounds. This should be included as a limitation of your study.**

We agree with the reviewer 2 with their point that the experimental design might have introduced order confounds. This comment was also one of the concerns of reviewer#1 (see point 1). We are thankful for this comment and as suggested we have elaborated this confound in the section Limitations (page 29, line 720 ff.).

8. **Page10, lines 193-195: I do not understand why all participants rated the products in the same sequence. This design choice introduces potential order effects and undermines randomization.**

We appreciate the comment regarding the trial sequence and understand that, particularly from a behavioral task perspective, a randomized design would have been preferable to avoid potential confounds, and we have therefore included this point in the revised manuscript (see section “Limitations”, page 29, line 726 ff.).We opted for a within-subject pseudo-randomized design due to the constraints of the event-related fMRI setup. Specifically, we implemented a sequence in which no condition was immediately repeated (we had a total of nine conditions: three frame types across three decision domains). This was crucial given the timing parameters of the task: each trial lasted approximately two seconds (response-dependent), and the jittered fixation cross also averaged around two seconds. Since the BOLD response is expected to peaks around six to eight seconds after stimulus onset and requires additional time to return to baseline, it was essential to ensure temporal separation between conditions to allow for accurate modeling of condition-specific neural activity. A fully randomized design could have led to consecutive trials of the same condition, introducing overlap in the hemodynamic responses and, consequently, errors in the statistical analysis. That said, we fully agree with the reviewer that a conditioned randomized design would still have been advantageous, particularly for behavioral analyses and we therefore as mentioned included it in the “Limitations” (see page 29, line 720 ff.).

9. **Page 13-14: Your current analyses do not take into account the hierarchical data structure (i.e., you average the responses across the 21 green, yellow, and red-framed products) for each dependent variable. Hence, you ignore trial level variability as well as potential differences between stimuli. Therefore, I would recommend to run multilevel analyses. In addition, multilevel models can deal better with missing data (which you very likely have as respondents have to answer quickly).**

We thank the reviewer for this important suggestion. In response, we conducted linear mixed-effects models for all key dependent variables: WTP, perceived healthiness, and perceived tastiness. These models account for trial-level variability by including random intercepts for both participants (id) and stimuli (product), and controls for between-subject and between-stimulus differences, thereby addressing the hierarchical nature of the data and the potential presence of missing responses. Overall, the results are consistent with the behavioral findings reported in the manuscript, indicating that including products as a random effect did not substantially alter the pattern of results. Therefore, we kept the original analysis, as was also preregistered. In the following, the results of the analysis can be found.

- WTP

The mixed model revealed significant main effects of color-coded frame (*F*(2, 60.1) = 5.74, *p* = .005) and Treatment (*F*(1, 4854.6) = 16.27, *p* < .001), as well as a significant interaction (*F*(2, 4854.6) = 4.75, *p* = .0087). Planned contrasts comparing Control vs. Treatment within each frame color yielded can be found under Table 5.

**Table 5.** Contrasts of WTP Between Treatment and Control Conditions Within Each Frame Color.

| Contrast | Estimate | SE | z | p |
| --- | --- | --- | --- | --- |
| Green - Control vs Treatment | 0.009 | 0.030 | 0.292 | .770 |
| Yellow - Control vs Treatment | 0.062 | 0.030 | 2.07 | .039 |
| Red - Control vs Treatment | 0.139 | 0.030 | 4.63 | < .001 |

These results indicate that Treatment significantly reduced WTP for red- and yellow-framed products, but not for green-framed products.

- Healthiness Perception

The model showed a significant main effect of color-coded frame (*F*(2, 60.0) = 12.92, *p* < .001) and a strong interaction with Treatment (*F*(2, 4840.2) = 113.39, *p* < .001). The main effect of Treatment was not significant (*F*(1, 4840.2) = 3.43, *p* = .064). The planned contrasts of healthiness perception can be seen in Table 6.

**Table 6.** Contrasts of Healthiness Perception Between Treatment and Control Conditions Within Each Frame Color.

| Contrast | Estimate | SE | z | p |
| --- | --- | --- | --- | --- |
| Green - Control vs Treatment | –0.436 | 0.048 | –9.03 | < .001 |
| Yellow - Control vs Treatment | 0.004 | 0.048 | 0.090 | .928 |
| Red - Control vs Treatment | 0.586 | 0.048 | 12.20 | < .001 |

These results show that the treatment effect strongly depended on frame color: participants rated red-framed products as significantly less healthy in the treatment condition, while the green frame led to higher perceived healthiness under treatment. No significant difference was found in the yellow condition.

- Tastiness Perception

The model revealed significant effects of color-coded frame (*F*(2, 60.1) = 3.83, *p* = .027), Treatment (*F*(1, 4848.4) = 12.07, *p* < .001), and a significant interaction (*F*(2, 4848.4) = 3.29, *p* = .037). The planned contrasts for the decision domain tastiness perception can be found under Table 7.

**Table 7.** Contrasts of Tastiness Perception Between Treatment and Control Conditions Within Each Frame Color.

| Contrast | Estimate | SE | z | p |
| --- | --- | --- | --- | --- |
| Green - Control vs Treatment | 0.001 | 0.065 | 0.021 | .983 |
| Yellow - Control vs Treatment | 0.158 | 0.065 | 2.41 | .016 |
| Red - Control vs Treatment | 0.234 | 0.065 | 3.58 | < .001 |

These findings suggest that tastiness ratings showed a significant decrease in the treatment condition for red and yellow-framed products, while no change was observed in the green condition.

10. **Page 13-14: In line with comment number 5, I am worried that the fact that you found that WTP and tastiness dropped in the color conditions may be partly explained by participant fatigue.**

We understand the reviewer’s concern and appreciate the feedback. We have now addressed this point in the Limitations section of the manuscript (see page 30, line 753 ff.). However, we consider participant fatigue is a minor explanation for the observed effects. Although we did not explicitly assess fatigue levels before and after the experiment, we examined reaction times as an indirect indicator. If fatigue had played a substantial role, we would expect either (a) accumulating longer reaction times across the task, indicating slower decision-making due to exhaustion, or (b) unusually short reaction times, which could suggest disengaged or hasty choices made without proper consideration. As shown in our response time analysis (see S3 File), a repeated-measures ANOVA revealed no significant interaction between treatment condition and frame color on response times. While participants did respond more quickly over time, their average reaction time remained above two seconds, suggesting that decisions were still deliberate. We interpret this pattern as a possible learning or task-familiarization effect, rather than a sign of fatigue. Moreover, if fatigue were amplifying the use of heuristics associated with the color-coded frame, we would expect to see this across all frame colors, including the green frame. However, no such effect was observed for green-labeled products in either WTP or tastiness ratings, which further argues against fatigue as a driving factor. Nevertheless, as we cannot entirely rule out this possibility, we have acknowledged it as a limitation in the revised manuscript (see page 30,line 752 ff).

11. **Page 13-14: I would suggest reporting effect sizes (cohen’s d) for the post hoc tests.**

We thank the reviewer for this helpful suggestion. In response, we have added the corresponding effect sizes (Cohen’s d) for all post hoc tests.

12. **It is important to rewrite the discussion. Now it is constantly mentioned that you studied whether labels impact decision values, this claim overstates the manipulation. A color-coded frame is not equivalent to a front-of-pack nutrition label.**

We agree with the reviewer, particularly as it builds on earlier feedback. We have revised the Discussion section accordingly. Nevertheless, based on the points raised in previous comments and our findings, our data supports that the color coded frame serves as FoPL. Thus, participants perceive the frame as conveying informational value rather than merely responding to color alone. Here, we would also like to refer to our Limitations (page 28, line 695 ff.), where we acknowledge that the color-coded frame is not the same as the label itself and mention the differences.

13. **Page 19, lines 383: You mention “it was hypothesized” though no hypothesis were included in the manuscript.**

We thank the reviewer for this point. As this comment is based on the first point of the reviewer, we would like to kindly refer to our answer there. We have adjusted the terminology for the hypotheses to be able to find our hypotheses more easily (see page 6, line 125 ff.).

14. **Page 19, lines 387-388: Why would you expect that the green frame would positively impact tastiness perceptions? Participants were informed upfront that the frames were health-related, so why would participants infer that a healthier product is also tastier? This is actually going against a large body of literature on the unhealthy = tasty intuition (Raghunathan, Naylor, & Hoyer, 2006). Reference: Raghunathan, R., Naylor, R. W., & Hoyer, W. D. (2006). The unhealthy= tasty intuition and its effects on taste inferences, enjoyment, and choice of food products. Journal of Marketing, 70(4), 170-184.**

We acknowledge the well-established unhealthy = tasty intuition (Raghunathan, et al., 2006) and are well aware of the study, which suggests that consumers often perceive unhealthy foods as more palatable. However, recent literature increasingly challenges this heuristic and highlights that the relationship between healthiness and tastiness is more complex.

While some individuals may indeed associate healthiness with reduced taste, several studies have found a positive relationship between perceived healthiness and tastiness (Haasova & Florack, 2019a; Haasova & Florack, 2019b; Jo & Lusk, 2018; Werle et al., 2013). For example, Haasova and Florack (2019a) conducted cross-national studies and consistently reported that healthier foods were rated as tastier, although the strength of this effect varied depending on product category and individual differences such as health interest, food pleasure orientation, and belief in the unhealthy = tasty heuristic. Cultural context also appears to play an important role. While the original study was conducted in the United States, studies with European and East-Asian subject pools have reported different patterns. Werle et al. (2013) showed that French consumers, for instance, tended to perceive healthy foods as tastier, while associating unhealthy foods with worse taste. Similarly, Jo and Lusk (2018) found that FoPL increased both perceived healthiness and tastiness of products among American, Chinese, and Korean consumers, indicating a positive link between these attributes. As our participant pool consisted of only German participants, we believe that this unhealthy = tasty intuition might not be applicable in our study. Moreover, our representative sample was controlled for their BMI whereas the participants in the study by Raghunathan and colleagues (2006) did not control obesity. Given recent advances related to the unhealthy = tasty intuition, we adopted the value-based decision-making model as a more robust theoretical framework for our hypotheses. We assumed that increases in the subjective valuation of a product, as measured by WTP, would be accompanied by corresponding shifts in the perceived value of its attributes. This assumption formed the basis of our preregistered hypothesis and guided the present study. In the *Introduction* section, we now make it clearer that the hypotheses are derived by the value-based decision-making model (see page 6, line 127 ff.)

15. **Page 13, line 173: I would start the sentence with “An ANOVA revealed…”**

We thank the reviewer for this helpful suggestion. We have implemented the recommendation in the Results section (“Behavioral data – Effects of the color-coded frame on WTP, healthiness perception, and tastiness perception”) on page 15, line 345.

16. **Small typo on page 14, line 274: η²partial = 46 should be η²partial = .46**

We thank the reviewer for noticing this typo. We have corrected it, and the value now reads as η²partial = .46 on page 15, line 347.

**Reference List**

Cadario, R., & Chandon, P. (2020). Which healthy eating nudges work best? A meta-analysis of field experiments. *Marketing Science*, *39(3),* 465-486. https://doi.org/10.1287/mksc.2018.1128

Enax, L., Hu, Y., Trautner, P., & Weber, B. (2015). Nutrition labels influence value computation of food products in the ventromedial prefrontal cortex. *Obesity, 23(4),* 786–792. <https://doi.org/10.1002/oby.21027>

Fehr, E., & Rangel, A. (2011). Neuroeconomic Foundations of Economic Choice—Recent Advances. *Journal of Economic Perspectives, 25(4)*, 3–30. <https://doi.org/10.1257/jep.25.4.3>

Haasova, S., & Florack, A. (2019a). Practicing the (un) healthy= tasty intuition: Toward an ecological view of the relationship between health and taste in consumer judgments. *Food Quality and Preference, 75,* 39-53. https://doi.org/10.1016/j.foodqual.2019.01.024

Haasova, S., & Florack, A. (2019b). Sugar labeling: How numerical information of sugar content influences healthiness and tastiness expectations. *PLoS One, 14(11)*, e0223510. <https://doi.org/10.1371/journal.pone.0223510>

Javaheri, N., Doehring, N., & Herrmann, M. (2023). Nudging Dietary Decisions with the Nutri-Score-an fMRI study. *PsychArchives.* https://doi.org/10.23668/psycharchives.13146

Jo, J., & Lusk, J. L. (2018). If it’s healthy, it’s tasty and expensive: Effects of nutritional labels on price and taste expectations. *Food Quality and Preference, 68*, 332-341. https://doi.org/10.1016/j.foodqual.2018.04.002

Knutson, B., & Genevsky, A. (2018). Neuroforecasting aggregate choice*. Current directions in psychological science, 27(2),* 110-115. <https://doi.org/10.1177/0963721417737877>

Raghunathan, R., Naylor, R. W., & Hoyer, W. D. (2006). The unhealthy= tasty intuition and its effects on taste inferences, enjoyment, and choice of food products*. Journal of Marketing, 70(4)*, 170-184. https://doi.org/10.1509/jmkg.70.4.17

Reeck, C., Wall, D., & Johnson, E. J. (2017). Search predicts and changes patience in intertemporal choice*. Proceedings of the National Academy of Sciences, 114(45),* 11890–11895. <https://doi.org/10.1073/pnas.1707040114>

Werle, C. O., Trendel, O., & Ardito, G. (2013). Unhealthy food is not tastier for everybody: The “healthy= tasty” French intuition. *Food Quality and Preference, 28(1)*, 116-121. https://doi.org/10.1016/j.foodqual.2012.07.007
