# Supplementary material for: From attributes to value: Neural correlates of a front-of-package label on food decision-making – An fMRI study
Source: PLoS One. 2025 Dec 5;20(12):e0336356. doi: 10.1371/journal.pone.0336356 (PMC12680182; doi:10.1371/journal.pone.0336356)
Supplement: Attachment — Submitted filename: Response to Reviewers_Round2.docx [file pone.0336356.s023.docx]

From,

Negin Javaheri

Department of Neuropsychology and Behavioral Neurobiology,

University of Bremen

To,

Dr. Ogawa

**Subject:** Rebuttal Letter for Manuscript PONE-D-25-19520

From attributes to value: neural correlates of a front-of-package label on food decision-making – an fMRI study

PLOS ONE

Dear Dr. Ogawa,

We sincerely appreciate the invitation to revise and resubmit our Manuscript PONE-D-25-19520 entitled " From attributes to value: neural correlates of a front-of-package label on food decision-making – an fMRI study" to PLOS One. We are happy to have satisfied most of the previous comments and are grateful for the additional comments provided by the second reviewer, that have helped us to clarify and improve the quality of our work. Below, we will provide a detailed response to each of his/her comments. For the sake of clarity, the reviewers’ comments are included in bold, followed by our responses in plain text. All changes made to the manuscript are attached as ‘Revised_Manuscript_PONE-D-25-19520_with_Track_Changes’, further we attach the manuscript without track mode as ‘Revised_Manuscript_ PONE-D-25-19520’. Please note that the page and line references refer to the revised manuscript without track changes.

Additionally, we made minor revisions to Fig. 1 (clarified that different products have different color-coded frames) and Fig. 3 (added the coordinates of the images), which we have also uploaded. Moreover, since we substantially revised the introduction, we have thoroughly updated the reference list as well.

Thank you for your time and consideration.

Best Regards,

Negin Javaheri

**Reviewer #2**

1. **I would kindly suggest formulating the hypotheses in a more formal and structured way. It would be helpful to number them and present them in a separate paragraph (e.g., H1, H2, etc.), so that you can then clearly refer back to them in the results section.**

We thank the reviewer for this helpful suggestion. In the revised manuscript, we have reformulated our hypotheses into a more formal and structured format, numbering them as H1–H3 and presenting them in separate paragraphs in the Introduction section (p. 5, lines 82-86; p. 7, lines 132-143). This revised section now clearly distinguishes between behavioral (H1) and brain activity derived hypotheses (H2–H3), which we refer back to in the Results (e.g., p. 15, line 295) and Discussion sections (e.g., p. 21, line 425). We have additionally changed the Figures (4-6) in a way that the hypothesized regions of the neural hypotheses (H2 and H3) are better detectable.

2. **Regarding my earlier third comment, I feel it has not been fully addressed. As I mentioned, your study examines an affective rather than a cognitive nudge. However, references on how affective nudges influence consumer behavior are still missing. Adding these would considerably strengthen the theoretical foundation of your paper.**

We thank the reviewer for reiterating this important point. We acknowledge that the color-coded frame shows a strong affective component and cannot be considered a purely cognitive nudge. In the revised manuscript, we now explicitly mention this aspect of the Nutri-Score in the introduction chapter (p. 4, lines 56–60) and note that effect sizes on effectiveness of the nudges on making healthier food decisions can vary between different nudge classifications. Additionally, in the theoretical background of the study we further elaborate the experimental conditions of the mentioned studies, where a Guideline daily amount (GDA) label (information-based label without color code) was compared with traffic light labels (information based label with color-grading) to make it more comparable with our study, where we also used traffic-light color grading in form of a frame around food items (p. 5, lines 94 ff.). We also clarify in the limitation section (p. 29, lines 624 ff.) that our manipulation emphasized the affective (color-based) component of the Nutri-Score while preserving its cognitive content. Importantly, all participants were informed about the meaning of the frame and the Nutri-Score information before the task and post-hoc analyses (see S4 File) confirm that the frames inferred the intended evaluative information. Further, we conducted an additional neuroforecasting study that is not part of the present study but clearly demonstrated that the neural activity of the color-coded frame conditions of our fMRI sample could forecast binary choices of food products with the Nutri-Score label of an independent sample. This data shows that our manipulation did not solely rely on an emotional response, even though affective processes might have contributed to decision-making, and that the color-coded frame was associated with Nutri-Score information. Finally, we refined the research question in the introduction chapter to explicitly state that the study investigates the impact of the Nutri-Score–derived color-coded frame on three key components of dietary decision-making at the neural level. We hope that together, these changes clarify the theoretical framing and address the reviewer’s concern.

3. **More generally, I think the theoretical background could benefit from a clearer structure. I would suggest starting with a discussion of behavioral responses to color-coded labels and frames, exploring this stream of research in more depth, and then formulating hypotheses (H1, H2, etc.) related to the behavioral aspects. Afterwards, you could discuss the literature on neural responses and also formulate formal hypotheses in this section. Following the same structure throughout the paper would make it much easier for readers to follow your argument.**

We thank the reviewer for this valuable suggestion. In the revised manuscript, we have substantially restructured the theoretical background to improve clarity and align with the proposed organization. Specifically, we now (i) first discuss behavioral evidence on the effects of color-coded labels and frames on food choice, healthiness perception, and tastiness perception, (ii) follow this section with a clearly formulated behavioral hypothesis (H1), and (iii) subsequently present the literature on neural responses before introducing the neural hypotheses. We have also revised the presentation of the neural hypotheses: rather than grouping them by decision domain, we now group them according to the two main pathways involved, the ventral reward pathway and the cognitive control pathway. This new structure also mirrors the organization of the Results section, thereby ensuring consistency throughout the paper and making it easier for readers to follow the argument from theory to results and discussion.

4. **Page 21, lines 412–414: I recommend nuancing the statement “which is consistent with prior research of how FoPL alter choices (2,19).” For the Nutri-Score in particular, findings are mixed: while some studies show that consumers tend to select more healthy products, they do not substantially modify their choices of unhealthy products (see below). van den Akker, K., Bartelet, D., Brouwer, L., Luijpers, S., Nap, T., & Havermans, R. (2022). The impact of the nutri-score on food choice: A choice experiment in a Dutch supermarket. Appetite, 168, 105664. De Temmerman, J., Heeremans, E., Slabbinck, H., & Vermeir, I. (2021). The impact of the Nutri-Score nutrition label on perceived healthiness and purchase intentions. Appetite, 157, 104995.**

We thank the reviewer for this helpful suggestion. In the revised manuscript, we have nuanced our interpretation of the findings to reflect that the color-coded frame primarily discouraged unhealthy choices rather than actively promoting healthier ones, contradicting previous findings (p. 21, lines 428 ff.). We have also explicitly acknowledged that prior evidence on the Nutri-Score report effects mainly on the selection of healthier products, and we refer to the respective studies (e.g. De Temmerman et al., 2021; van den Akker et al., 2022).

5. **I recommend adding the multilevel results to the appendices, as these analyses are, in my opinion, more appropriate and provide a more accurate representation than the current approach. Including them as supplementary material would strengthen the manuscript and allow readers to better assess the robustness of the findings.**

We appreciate the reviewer’s suggestion and have implemented the multilevel results to the appendices (File S6). In addition, we refer to the multilevel analysis in the “Behavioral Data” section of the discussion (p. 21, line 427 ff.).
